# Novel targets, treatments, and advanced models for intracerebral haemorrhage

**DOI:** 10.1016/j.ebiom.2022.103880

**Published:** 2022-02-12

**Authors:** Marietta Zille, Tracy D. Farr, Richard F. Keep, Christine Römer, Guohua Xi, Johannes Boltze

**Affiliations:** a Department of Pharmaceutical Sciences, Division of Pharmacology and Toxicology, University of Vienna, UZA II, Althanstr. 14, Vienna 1090, Austria; b School of Life Sciences, Physiology, Pharmacology, and Neuroscience Division, Medical School, University of Nottingham, Nottingham NG7 2UH, UK; c Department of Neurosurgery, University of Michigan, Ann Arbor, MI 48109-2200, USA; d Max Delbrück Center for Molecular Medicine in the Helmholtz Association, The Berlin Institute for Medical Systems Biology, Berlin 13125, Germany; e School of Life Sciences, The University of Warwick, Gibbet Hill Campus, Coventry CV4 7AL, UK

**Keywords:** Animal models, Brain haemorrhage, Aetiology, Haematoma, Inflammation, Recovery

## Abstract

Intracerebral haemorrhage (ICH) is the second most common type of stroke and a major cause of mortality and disability worldwide. Despite advances in surgical interventions and acute ICH management, there is currently no effective therapy to improve functional outcomes in patients. Recently, there has been tremendous progress uncovering new pathophysiological mechanisms underlying ICH that may pave the way for the development of therapeutic interventions. Here, we highlight emerging targets, but also existing gaps in preclinical animal modelling that prevent their exploitation. We particularly focus on (1) ICH aetiology, (2) the haematoma, (3) inflammation, and (4) post-ICH pathology. It is important to recognize that beyond neurons and the brain, other cell types and organs are crucially involved in ICH pathophysiology and successful interventions likely will need to address the entire organism. This review will spur the development of successful therapeutic interventions for ICH and advanced animal models that better reflect its aetiology and pathophysiology.

## Introduction

Intracerebral haemorrhage (ICH) is caused by a loss of vascular integrity leading to bleeding within the brain parenchyma. ICH accounts for ^∼^28% of all strokes and has the highest mortality rates among all stroke types.^1^ ICH also contributes the largest proportion of disability-adjusted life-years of all neurologic disorders.^2^ Despite advances in medical care, the mortality and morbidity rates remain high and effective treatment options are lacking. This review gives an update on emerging targets and treatments for spontaneous (nontraumatic) ICH focusing on the past three years and attempts to uncover novel research avenues including filling gaps in animal modelling.

## Aetiology of ICH

**Hypertension** is the most common risk factor of spontaneous ICH, exhibited by up to 70% of ICH patients and antihypertensive treatment significantly reduces ICH risk. Animal models may help to reveal underlying cellular and molecular mechanisms of vascular instability prior to first ICH occurrence. Mouse models of hypertension-related spontaneous ICH are available (8% high-salt diet + the nitric oxide synthase inhibitor L-NAME in drinking water on a double transgenic background of renin and angiotensinogen overexpression^3^; angiotensin II pump infusion + L-NAME in drinking water + injection of angiotensin II or norepinephrine in C57BL/6 mice).^4^ These models better reflect the more complex rupture of a blood vessel than what is modeled by cerebral collagenase injection.^5^ Major drawbacks are the relatively small ICH volume and thus comparatively short haemorrhage resolution time, as well as the unpredictable time of ICH onset.

**Smooth muscle cell degeneration** at cerebral arterioles is common in cerebral small vessel disease that frequently underlies ICH. It is intuitive to hypothesize that weakened arterioles eventually burst when exposed to high perfusion pressure, most commonly in deep brain regions where penetrating arterioles are in close proximity to the circle of Willis. However, smooth muscle cell degeneration alone may not be sufficient to induce blood vessel rupture. In mice with a mutation in the α1 chain of collagen type IV (COL4A1), a model of spontaneous ICH, hypermuscularization in the transitional segment between arterioles and capillaries led to an increase in intravascular pressure in the upstream arteriole that bursts at sites of smooth muscle cell loss.^6^ Similarly, combining angiotensin II-induced hypertension with low serum uric acid levels worsened the disruption of the smooth muscle cell-elastin contractile unit in cerebral vessels and ICH progression in mice.^7^ This highlights the importance of using spontaneous models of ICH for investigating aetiology.

**Cerebral amyloid angiopathy** (CAA) is characterised by amyloid β deposits in leptomeningeal and cerebral blood vessel walls decreasing vessel diameter and leading to microaneurysms which can cause ICH. Patients with CAA-related ICH also have a greater risk for ICH recurrence.^8^ CAA is mainly (80%) sporadic, but hereditary forms exist. Only the hereditary forms can be sufficiently modelled in animals (Swedish K670N/M671L and Dutch/Iowa E693Q/D694N mutations in the amyloid precursor protein^9^), which to some extent can also mimic sporadic CAA. Whereas these transgenic mice display cerebral microbleeds, preclinical models of CAA leading to large ICH are lacking. Promising therapeutic approaches have been developed around amyloid β clearance.^10^

**Oral anticoagulant** use increases the risk of ICH 7- to 10-fold. However, that risk is reduced in patients treated with direct oral anticoagulants (DOACs) compared to warfarin (**Supplementary Table**).^11^ Preclinical studies using collagenase- or laser-induced ICH in rodents have replicated the benefit of DOACs^12^ and the effectiveness of reversing anticoagulation was examined in animal models.^13^ In mice subjected to cerebral microbleeds, warfarin promoted deadly ICH, whereas DOACs increased microbleed burden without triggering long-term cognitive impairment.^14^ Whether anticoagulant treatment provokes ICH by aggravating existing microbleeds in humans remains unknown.

**Vascular malformations** including cerebral cavernous (CCMs) and arteriovenous malformations (AVMs) are major risk factors for ICH. CCMs are clusters of abnormal venous capillaries. There is no structural support of blood vessels from smooth muscle cells making CCMs vulnerable to rupture. Although most CCMs develop sporadically, several mutations causing CCMs have been identified (KRIT1/CCM1, malcavernin/CCM2, PDCD10/CCM3) and respective mouse models are available.^15^^,^^16^ AVMs are congenital entanglements of arterial vessels directly connected to the venous system without an intermediate capillary bed. Excessive vascular endothelial growth factor (VEGF) signalling is believed to promote AVMs, and Ras plays an important role for physiological VEGF signalling. Mutations in genes involved in the RAS/MAPK pathway such as KRAS have been observed in AVM endothelial cells in human patients.^17^ A mouse model of controllable Ras overactivation in endothelial cells has been created, in which cerebral AVMs and spontaneous ICH are observed.^18^

**Hypercholesterolaemia** is associated with an increased risk for cardiovascular diseases, but decreases the risk for ICH in both sexes.^19^ However, hypercholesterinaemia can aggravate neuroinflammatory reactions after ICH similar to hypertension. An increased recruitment of neutrophils and monocytes is observed in dyslipidaemic mice, leading to poor functional outcome and exacerbated perihaematomal oedema.^20^ Because of the high prevalence of hypercholesterinaemia in humans, further investigations in dyslipidaemic ICH models are warranted.

## Haematoma

**Haematoma expansion** occurs in 20–40% of patients over the first day after ICH. Haematoma volume is a major determinant of outcome, and clinical trials have focused on limiting haematoma expansion. Phase III clinical trials (INTERACT2, NCT00716079; ATACH-II, NCT01176565) have investigated pharmacological blood pressure lowering, including intensive approaches, after ICH (**Supplementary Table**). Current recommendations strongly suggest that elevated blood pressure should be treated as early as possible in patients with acute ICH.^21^ A recent phase II trial (ICH-ADAPT II, NCT02281838, **Supplementary Table**) aims to identify the benefit of aggressive vs. conservative blood pressure lowering. It should be noted that studies focused on reducing blood pressure to limit secondary haematoma expansion after ICH have focused on reducing systolic or mean arterial blood pressure. It may be that fluctuations in pressure and blood flow play a role in inducing continued bleeding. Other trials to limit haematoma expansion include using haemostatic agents (tranexamic acid (TRANSACT, NCT03044184; STOP-MSU, NCT03385928) and recombinant Factor VIIa (FAST, NCT00127283; FASTEST, NCT03496883, **Supplementary Table**). Preclinical studies on haematoma expansion are hampered by available models. Yet, intracerebral injection of collagenase or autologous blood does produce a haematoma that initially expands and that can be exacerbated by hypertension and hyperglycaemia.^5^^,^^22^ However, there are concerns that the underlying mechanisms (e.g., gradual degradation of the endothelial basement membrane) do not reflect those occurring in ICH patients. A model using liquid polymer gel that coagulates on contact with tissue has recently been devised to create a mass that causes secondary bleeding, the extent of which is blood pressure-dependent.^23^ However, this model is lacking haemolysis-induced toxicity. Nevertheless, it may be useful for comparing different methods for limiting haematoma expansion.

**Figure 1:**
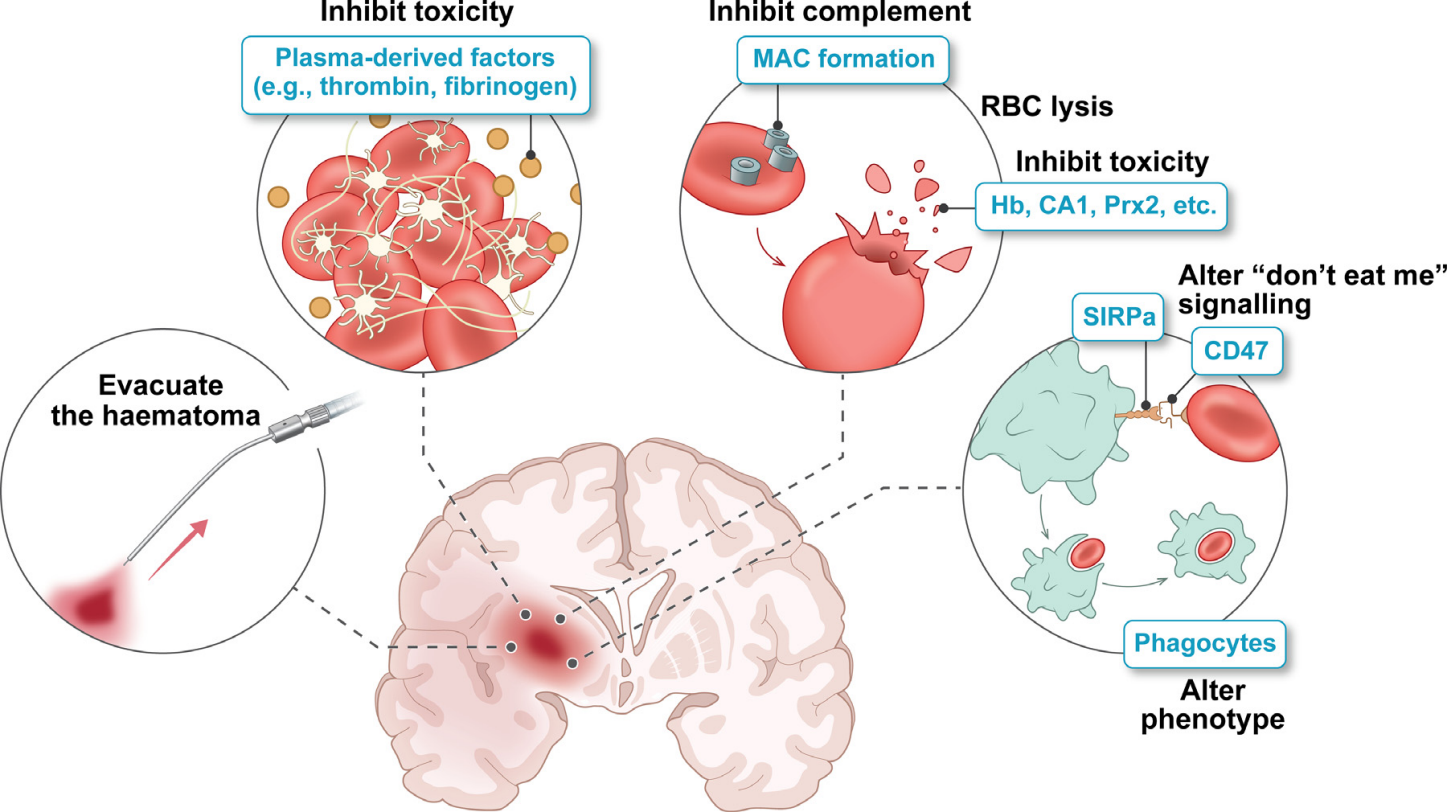
The haematoma as a therapeutic target in ICH. The haematoma can cause brain injury via mass effect and the release of potentially toxic, plasma- or erythrocyte-derived factors. Surgical haematoma evacuation and accelerating endogenous haematoma clearance via phagocytosis are potential approaches, as is delaying erythrocyte lysis and inhibiting deleterious effects of clot-derived factors. RBC, red blood cell; MAC, membrane attack complex; Hb, haemoglobin; CA1, carbonic anhydrase 1; Prx2, peroxiredoxin-2; SIRPα, signal regulatory protein alpha.

The impact of physical **haematoma evacuation** (Figure 1) has been examined in many clinical trials with, as yet, no evidence of improved neurological outcome (STICH II, ISRCTN22153967; MISTIE-III, NCT01827046, **Supplementary Table**).^24^^,^^25^ An alternate approach may be to accelerate **endogenous haematoma resolution**. Currently, in rodents, multiple agents such as peroxisome proliferator-activated receptor-γ and retinoid X receptor agonists have been shown to alter microglia/macrophage phenotype, enhance phagocytosis, speed haematoma clearance, and improve neurological outcome.^26^^,^^27^ Endogenous IL-4/Stat6 signalling is important in regulating haematoma resolution. Intranasal delivery of IL-4 nanoparticles also speeds resolution and improves neurological outcomes.^28^ An alternate approach is to block ‘don't-eat-me’ signals expressed on erythrocytes that normally suppress phagocytosis (e.g., using a CD47 antibody^29^). Whereas rodent ICH models have provided insight into mechanisms regulating endogenous haematoma clearance, studies on gyrencephalic species with larger brain sizes (e.g., pig, sheep) are needed to examine the effects of haematoma size and species physiological differences on endogenous clearance. Translation to the clinic may require a combination with physical evacuation to debulk the haematoma with accelerated endogenous clearance mechanisms to remove residual haematoma.

**White matter (WM) injury with demyelination and axonal degeneration** commonly occurs in human ICH. Despite this, WM injury has received less attention in preclinical ICH models in part because rodents have limited WM. Evidence indicates that ICH induces WM injury via multiple mechanisms including mechanical injury, oxidative stress (in part haemoglobin-/iron-mediated), neuroinflammation, excitotoxicity, and blood-brain barrier (BBB) disruption.^30^ Changes in the axonal cytoskeleton after ICH not only impact the physical structure of the axon but also mitochondrial transport and function leading to degeneration.^31^ Recent studies have suggested that the inhibition of histone deacetylases (HDACs) with scriptaid or conditional knockout of HDAC2 in microglia can reduce ICH-induced neuroinflammation and WM injury in mice.^32^ Other studies have targeted neuroinflammation with the antibiotic and inhibitor of microglial activation minocycline in piglets,^33^ and the sphingosine-1-phosphate receptor modulator FTY720/fingolimod in mice.^34^ Both approaches reduced WM injury. It should be noted that fingolimod is currently undergoing clinical testing for ICH (FITCH, NCT04088630, **Supplementary Table**). A pilot study on minocycline (MACH, NCT01805895, **Supplementary Table**) demonstrated that 400 mg of minocycline were safe and resulted in neuroprotective serum concentrations.^35^ Further clinical trials are needed to demonstrate the efficacy of minocycline in treatment of ICH.

**Cell death** is a hallmark of ICH. Whereas the haematoma causes immediate damage to cells, clot-derived breakdown products induce cell death hours to days after the initial bleed. Importantly, different cell death subroutines occur after ICH, including autophagy, necroptosis, and ferroptosis.^36^^,^^37^ **Ferroptosis** is an iron-dependent, non-apoptotic form of regulated cell death that is driven by **lipid peroxidation** and critically depends on glutathione peroxidase 4 (GPX4). Lipid peroxidation has been demonstrated in animal models of ICH.^38^^,^^39^ Knockout of 5-lipoxygenase improved functional recovery and N-acetylcysteine inhibited its toxic arachidonic products.^38^ Furthermore, the lipid peroxidation inhibitors ferrostatin-1 and liproxstatin-1 reduced neurological deficits, memory impairment, brain atrophy, lesion volume, and neuronal cell death in collagenase and autologous blood infusion models in mice.^40^^,^^41^ Increasing the expression of selenoproteins including GPX4 by selenium supplementation or a brain-penetrant selenopeptide abrogated ferroptosis and improved functional outcome in collagenase-induced ICH in mice.^42^ In ICH patients, ferroptotic gene expression is increased,^41^ but studies demonstrating functional benefit of anti-ferroptotic drugs for patients are still required. It should be noted that a recent analysis of the iDEF trial (NCT02175225, **Supplementary Table**) indicated a benefit of deferoxamine in patients with moderate (10–30 ml) haematomas.^43^

Besides neurons, **brain endothelial cells** also undergo cell death after ICH.^44^ However, the underlying pathways are not yet fully elucidated. Endothelial dysfunction and BBB disruption have been demonstrated in collagenase and autologous blood infusion models of ICH in mice^45^ as well as in sheep in regions distant from the haematoma.^46^ The impairment of the BBB in ICH leads to brain oedema formation, the infiltration of immune cells, and the leakage of neurotoxic, pro-inflammatory, and vasoactive molecules.^47^

ICH-induced cytotoxic and vasogenic brain oedema are intimately connected to parenchymal cell injury and vascular injury/BBB disruption. Currently, **oedema treatments** are generally limited to hyperosmotic solutions (mannitol/hypertonic saline) or hyperventilation. Both types of oedema are associated with a brain build-up of ions (e.g., Na^+^ and Cl^−^) and there has been considerable interest in the use of glibenclamide, an inhibitor of the sulfonylurea receptor 1-regulated ion channels, for reducing perihaematomal oedema including a recently completed trial in ICH (GATE-ICH, NCT03741530, **Supplementary Table**). It should be noted that there is some disagreement in the preclinical literature about the effectiveness of glibenclamide in ICH.^48^^,^^49^

## Inflammation in ICH

**Figure 2:**
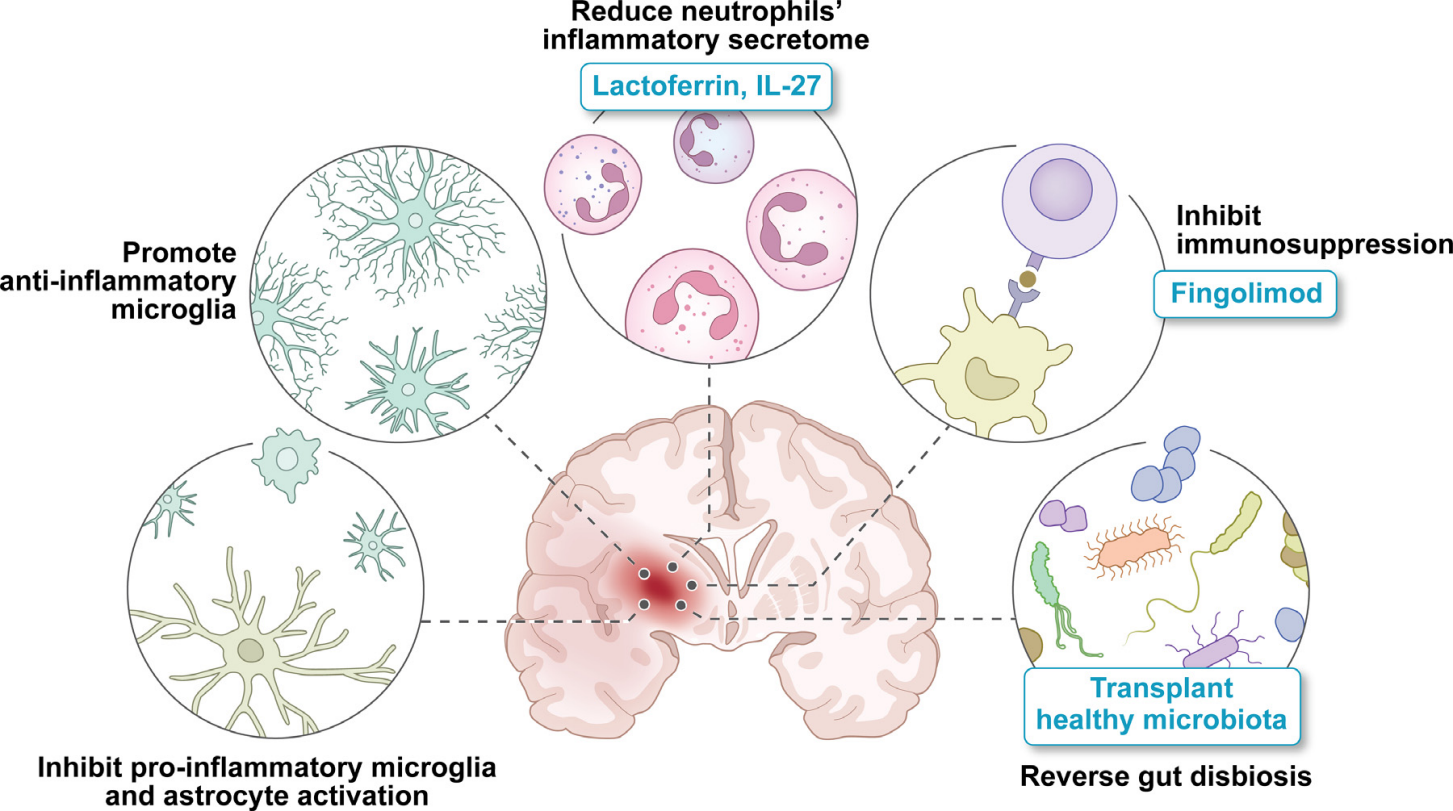
Inflammation as a therapeutic target in ICH. The haematoma induces local inflammatory events, mediated by microglia and astrocytes, as well as acute systemic inflammation, resulting in the recruitment of immune cells to the brain. The injured CNS then triggers systemic immunosuppression and changes in the gut-brain axis.

**Inflammatory** events can be triggered by intraparenchymal blood (Figure 2), and partly resemble that in ischaemic stroke. They are, however, incompletely investigated and their temporal profile is not well understood. Local microglia and astrocytes are first responders and their proinflammatory activation promotes circulating immune cell influx, predominantly of macrophages. There is an increased release of inflammatory cytokines (e.g., interleukin-1β, tumor necrosis factor), free radicals, and chemokines. This attracts and activates lymphocytes. Importantly, these processes also contribute to perihaematomal oedema formation and potentially ICH growth by compromising BBB integrity.^50^ Most ICH models in immunocompetent animals allow investigating post-ICH inflammation impact and kinetics, but more specialised models exhibiting relevant comorbidities may provide better insights into inflammation post ICH.

**Proinflammatory microglia/macrophages** (often referred to as the M1 subtype) are considered promising therapeutic targets in early ICH as they drive neuroinflammation that is mainly associated with inferior outcome. Beneficial effects were observed with minocycline, a central nervous system (CNS)-penetrant tetracycline with inhibitory activity on pro-inflammatory microglia/macrophages, in rodent ICH models, but early-stage clinical studies were inconclusive.^51^ At later stages, anti-inflammatory (M2) microglia/macrophage subtypes exert beneficial effects for instance by contributing to haematoma removal as well as perihaematomal oedema resorption. M2 microglia/macrophages also promote local white matter integrity, and even exert neuroprotective effects. Thus, an alternative approach with a potentially wider therapeutic time window would be timely, sub-acute promotion of anti-inflammatory microglia/macrophages.^52^ However, it is important to stress that neuroinflammation after ICH is a complex and continuous rather than dichotomous process that can have both detrimental and beneficial effects depending on the respective timepoint and pathobiological process affected. This should be taken into thorough consideration when developing precise experimental intervention strategies targeting neuroinflammation.

Inflammation and cytotoxicity driven by **neutrophils** are generally thought to exacerbate ICH injury, but neutrophils also exert beneficial and protective functions. For instance, the secretion of iron-scavenging lactoferrin can contribute to perihaematomal oedema reduction and increased haematoma clearance. Interleukin 27 reduces the amount of inflammatory/cytotoxic products in the neutrophil secretome and this increases the production of iron scavengers, thus shifting the balance in neutrophil action towards beneficial and protective effects.^53^ To note, this is a systemic process as the shift in the neutrophil secretome is induced in the bone marrow. Fusing human lactoferrin with the Fc domain of human IgG resulted in a molecule with a long plasma half-life and superior therapeutic outcome.^54^

To counterbalance inflammation, the injured CNS induces systemic **immunosuppression**. The overactivation of the sympathetic and parasympathetic nervous system in ICH results in spleen shrinkage and rapid lymphopenia. The degree of spleen shrinkage in ICH patients correlates with haematoma size, highlighting the link between ICH severity and immunosuppression, which in turn, predicts the likelihood of infections and impacts long-term outcome.^55^ Whereas the numbers of circulating (CD4^+^) T- and natural killer (NK) cells are decreased,^55^ there is a prominent invasion into the brain.^56^^,^^57^ Immunosuppression after ICH has been investigated in rodent ICH models using cerebral injection of autologous blood^58^ or collagenase,^59^ revealing programmed death ligand 1 (PD-L1) and metoprolol as potential therapeutic options. However, haematoma location and size vary considerably in patients and may influence how the haematoma regulates immune responses after ICH. Hence, there is a need for future models resembling the clinical situation more closely. Of note, the immunosuppressive effects of fingolimod (FTY720), currently in clinical trial for ICH treatment (FITCH, NCT04088630, **Supplementary Table**), rely mainly on inhibiting helper (CD4^+^) and effector (CD8^+^) T cells as well as CD19^+^ B cells.^60^ Hence, post-ICH immunosuppression should be considered when designing immunomodulatory interventions for ICH.

Modulating immune cells in the CNS also affects the microbiome via the **gut-brain-axis**. The microbiome is well-studied in several CNS pathologies, including ischaemic stroke and plays a role in neuroinflammation, neuroplasticity and autoimmunity. However, little is known about the cross-talk between the gut and the brain in ICH. In collagenase-induced ICH in mice, reduced gastrointestinal motility and microbiota dysbiosis have been demonstrated, and adversely affected outcome. Recolonising ICH mice with healthy microbiota has shown promising results.^61^ It would be important to further investigate microbiome changes in ICH patients as well as microbiome transplantations. A human stool bank for healthy microbiota already exists (openbiome.org) and would be a powerful tool to leverage.

## ICH sequelae and improving recovery

**Cardiac complications** are common after ICH and higher heart rate variability in the acute phase is associated with poorer 3-month outcomes.^62^ Preclinical studies using autologous blood or collagenase-induced ICH in mice have replicated cardiac complications^59^^,^^63^ and metoprolol reduced cardiac damage by abrogating sympathetic overactivation in addition to its immunosuppressive effects.^59^ Furthermore, splenectomy reduced cardiac dysfunction along with improving neurological outcome after autologous blood-induced ICH in mice.^64^ Due to their prevalence, cardiac complications should be assessed when testing novel therapeutic interventions preclinically.

**Enhancing post-ICH recovery** is an important aspect in therapeutic research. There is a plethora of well-established tests of motor, sensory, and cognitive functions for rodent ICH models.^65^ However, evidence for cognitive deficits is limited and spontaneous recovery of motor function and compensatory mechanisms in rodents may overestimate the impact of therapeutic interventions after brain injury; some tests may not be able to discriminate between these effects. Sensitive tests such as automated gait assessment or kinematic measures can provide valuable insights into functional recovery.^66^ Interestingly, it seems that rehabilitation strategies predominantly focusing on gross motor function may not be optimal in rodents,^67^ an important parallel to human patients.^68^ However, more complex neurorehabilitation strategies are under-investigated for ICH. A combined application of enriched environment and task-specific motor training showed improved outcome in a rat model of striatal ICH more than a decade ago,^69^ but more detailed research on optimal neurorehabilitation strategies is currently missing. It is known though that very early and intense rehabilitation can even impair functional recovery. This was shown in the phase III A Very Early Rehabilitation Trial after stroke (AVERT) which enrolled both patients with ischaemic and haemorrhagic stroke.^70^ Thus, timing and intensity of rehabilitation strategies requires further research. Interestingly, ICH location also impacts functional recovery. For instance, long-lasting deficits were observed after ICH in the internal capsule as compared to striatal lesions, despite a smaller lesion volume.^71^ ICH models targeting the internal capsule may therefore be well suited to investigate advanced rehabilitation or even restorative strategies. Detailed imaging protocols can be applied in ICH models^46^ and may reveal valuable morphological information related to functional recovery.

Depressive symptoms following ICH are common. **Post-ICH depression** may occur in up to 20% of patients and is associated with poorer long-term outcomes. Whereas treating depression is a priority, there is also an increased risk of secondary events associated with selective serotonin reuptake inhibitors.^72^ Furthermore, there has been interest in taking advantage of pleiotropic regenerative effects that drugs such as fluoxetine may offer. Three recent randomized clinical trials (FOCUS, ISRCTN83290762; AFFINITY, ACTRN12611000774921; EFFECTS, NCT02683213, **Supplementary Table**) assess fluoxetine for stroke recovery and all included ICH patients. They demonstrated that, whereas post-stroke depression was decreased, the risk of bone fractures and hyponatremia was increased and functional outcome was not improved. It is clear that post-ICH depression represents an important unmet need and novel treatments may require improved knowledge of the underlying mechanisms. This effort is hampered by the sparsity of preclinical research. Some studies suggest rodents with ICH exhibit deficits in the elevated plus maze, sucrose preference, tail suspension, open field, and forced swim tests,^73^^,^^74^ but others have failed^75^ resulting in uncertainty whether animal models accurately reflect cognition and depression.^66^ Recent studies have investigated genetic links between ICH and depression^76^ and the results support the monoaminergic hypothesis, as well as the idea that pro-inflammatory cytokines may stimulate the hypothalamic pituitary adrenal axis.^77^

## Emerging therapeutic approaches

**Small RNAs** (size <200 nucleotides) are readily accessible in body fluids. The most abundantly investigated in ICH diagnosis and therapy are micro-RNAs (miRNAs, 20–25 nucleotides). Their expression profile not only distinguishes ICH patients from healthy controls but also ischaemic stroke and subarachnoid haemorrhage patients, which makes them valuable diagnostic (e.g., miR-124-3p) and prognostic markers (e.g., miR-130a).^78^ Dysregulated miRNAs and their putative mRNA targets are commonly associated with pathways regulating neuroinflammation, cell death, vascular smooth muscle and focal adhesion.^79^ Normalising imbalanced miRNA levels in ICH using **miRNA mimics or antagomirs** leads to improved outcome after ICH in animals and *in vitro* models, including in models of the BBB. In addition, miRNA mimics and antagomirs in ICH therapeutics commonly have anti-inflammatory functions, reduce perihaematomal oedema and haematoma size (miR130a,^78^ miR-223,^78^ miR-194-5p,^80^ miR-152,^81^ miR-7-5p^79^), regulate BBB permeability (miR-130a,^78^ miR-27a,^82^ miR-126-3p^83^), promote neuronal survival (miR-27a,^82^ miR-152^81^), foster stem cell proliferation and migration, and improve endothelial function (miR-195^84^). This suggests that miRNA-based therapies can be successful in improving ICH outcome in the clinical context when administered after ICH if appropriate delivery can be achieved.

Whereas the role and therapeutic potential of miRNAs is actively investigated, the potential of **other small RNAs** is relatively unexplored. These small RNAs include PIWI-interacting RNAs (piRNAs), transfer RNA derived small RNAs (tsRNAs), small nuclear RNAs (snRNAs), small nucleolar RNAs (snoRNAs), and small Cajal body RNAs (scaRNAs). Seven tsRNAs have been demonstrated to be significantly changed in a rat model of collagenase-induced ICH and were involved in pathways participating in the oxidative stress response, endocytosis, and the regulation of G protein-coupled receptor signalling.^85^ Emerging clinical data highlights the differential expression of ribosomal and tRNA-derived fragments^86^ as well as snoRNAs^79^ in ICH compared with ischaemic stroke patients and healthy controls, respectively. The role of these dysregulated small RNAs in ICH is a field for future extensive studies and carries potential to open new avenues in understanding ICH pathobiology and developing ICH treatments.

**Exosomes**, also known as extracellular vesicles, are important mediators of regenerative mechanisms and exert therapeutic impact similar to that of regenerative cell populations. They are increasingly described in neurodegenerative disease, but knowledge in ICH is limited. Importantly, there is preliminary evidence for exosome-mediated anti-inflammatory mechanisms in mice^87^ and humans.^88^ Exosomes are frequently derived from mesenchymal stem cells (MSCs) as these cells are known to exert beneficial effects after ICH which are believed to be at least partly mediated by exosomes. Moreover, obtaining exosomes from MSCs is a well-established procedure. Specifically, exosomes obtained from bone marrow MSCs enriched with miR-146a-5p inhibited neuronal cell death and microglial M1 polarization compared to exosomes without enrichment.^89^ Exosomes from miR-19b-3p-mimic transfected adipose-derived stem cells were demonstrated to abrogate post-ICH ferroptosis in comparison to negative control mimic.^90^ Systemic delivery of miR-133b containing exosomes compared to miR-control reduced neurodegeneration by inhibiting RhoA and activating ERK1/2/CREB pathway even when administered 72 h after ICH,^91^ suggesting a clinical potential for this miRNA or exosomes as a delivery agent. A thorough characterisation of exosomes, including their cellular origin and content in the context of ICH will be important, and future research will also have to investigate therapeutic effects of exosomes derived from other (stem) cell populations.

**Nanoparticle**-based treatments, like many ICH-targeting therapies, have focused on mitigating pathogenic processes associated with the breakdown of blood in the brain. The most widely used approach has been to use nanoparticles to provide anti-oxidant benefits. Several nanoparticles have inherent anti-oxidant properties. For example, cerium oxide nanoparticles improved outcome in rodent models of ICH, with and without conjugation to polyethylene glycol (PEG), by reducing inflammatory activity and perihaematomal oedema formation.^92^^,^^93^ Several nanoparticle formulations are also amenable to functionalisation and/or modification to deliver therapeutic payloads. PEGylated hydrophilic carbon cluster nanoparticles have been bound to the iron chelator deferoxamine to further target ICH.^94^ Polymer-based nanoparticles remain popular as they are straightforward to manufacture, have multiple surface modification strategies, and are relatively stable. They have been modified to deliver oxidative therapies such as resveratrol^93^ and edaravone. The latter has potential for more rapid translation as edaravone has been used to treat ICH. Edavarone-containing nanoparticles further reduced perihaematomal oedema in patients with haematoma removal when compared to edaravone alone.^95^

## Limitations of animal models

As outlined above and summarized in Table 1, animal models have been used successfully to investigate some of the aetiological and pathophysiological mechanisms of ICH, but all have limitations. There are indeed limitations that are common across animal models, such as an extraordinary spontaneous recovery of sensorimotor function in the rodent, as well as limited evidence for cognitive deficits. While translational validity is an important consideration, no model perfectly recapitulates the complex aetiology of spontaneous ICH in humans. Models should always be chosen with this in mind according to the specific research question. Furthermore, the inclusion of multiple comorbidities within models (including hypertension, dyslipidaemia, diabetes, arteritis) is recommended in order to better reflect the clinical scenario, and when testing new therapeutic approaches, the use of at least two models reduces considerations surrounding limitations and increases security that mechanisms of interest are widely applicable.^96^ Moreover, age and sex should be more often considered in animal studies as they impact the pathophysiology and clinical outcomes.^97^^,^^98^ In addition to behavioural/functional outcome assessments, non-invasive imaging methods such as magnetic resonance imaging may give further and longitudinal insights into the underlying mechanisms such as white matter injury, axonal degeneration, and haematoma expansion as well as when assessing potential treatment candidates. Thorough outcome analysis requires sufficient time to allow the lesion to be finally organized and for potential improvements to plateau. Post-injury/-treatment surveillance time should be at least 3 weeks according to the Stroke Therapy Academic Industry Roundtable (STAIR) guidelines for preclinical research in ischaemic stroke using behavioural and structural or histological endpoints.^99^ Similar post-injury surveillance times are recommended for ICH.

**Table 1:** ICH animal models to address aetiological and pathophysiological mechanisms and their limitations. AVM, arteriovenous malformations; BBB, blood-brain barrier; CAA, cerebral amyloid angiopathy; CCM, cerebral cavernous malformation; COL4A1, α1 chain of collagen type IV; ICH, intracerebral haemorrhage; L-NAME, N^ω^-nitro-L-arginine methyl ester.

(A) Rodent models	
Model	Aetiological or pathophysiological mechanisms addressed, translational value
Autologous blood injection	- Endogenous haematoma clearance^26^^,^^28^^,^^29^^,^^53^ - White matter injury and axonal degeneration^30^^,^^100^ - Cell death^29^^,^^51^^,^^101^^,^^102^ - Perihaematomal oedema^49^^,^^51^^,^^53^^,^^101^^,^^102^ - BBB impairment^22^^,^^45^^,^^47^^,^^49^ - Inflammation^28^^,^^49^^,^^51^, ^52^, ^53^^,^^101^, ^102^, ^103^ - Immunosuppression^58^ - Cardiac complications^63^^,^^64^ - Can be combined with comorbidities: angiotensin II infusion + hyperglycaemia (incl. haematoma expansion),^22^ hyperglycaemia^102^
Collagenase injection	- Anticoagulation^12^^,^^13^ - Endogenous haematoma clearance^26^, ^27^, ^28^ - White matter injury and axonal degeneration^30^ - Cell death including ferroptosis^37^^,^^38^^,^^40^, ^41^, ^42^^,^^104^ - Perihaematomal oedema^27^^,^^48^ - BBB impairment^20^^,^^22^^,^^45^^,^^47^^,^^51^ - Inflammation^20^^,^^51^^,^^52^^,^^103^^,^^104^ - Immunosuppression^59^ - Gut-brain axis^61^ - Cardiac complications^59^ - Can be combined with comorbidities: dyslipidaemia,^20^ streptozotocin-induced diabetes,^104^ spontaneously hypertensive animals (incl. haematoma expansion)^5^
Injection of blood components	- Axonal degeneration^105^ - Cell death^51^ - Inflammation^51^ - White matter injury^106^
Laser-induced rupture of vessels	- Anticoagulation^107^ - Microbleeds^107^
Cyclodextrin nanoparticle injection	- Anticoagulation^14^ - Microbleeds^14^
Liquid polymer gel	- Hypertension (with additional phenylephrine injection)^23^ - Anticoagulation (with additional anticoagulant injection)^23^ - Mass effect, haematoma expansion^23^
High salt diet + L-NAME in drinking water in mice overexpressing human renin and angiotensinogen	- Reasonable modelling of the clinical situation - Spontaneous ICH - Hypertension as a risk factor for ICH^3^
Chronic angiotensin II infusion + L-NAME in drinking water + acute angiotensin II injection in mice	- Reasonable modelling of the clinical situation - Spontaneous ICH - Hypertension as a risk factor for ICH^4^ - White matter alterations^108^ - BBB impairment^108^ - Inflammation^108^ - Cognitive deficits and depression-like behaviour^108^
Chronic Angiotensin II infusion + L-NAME in drinking water + low serum uric acid levels in mice	- Reasonable modelling of the clinical situation - Spontaneous ICH - Hypertension as a risk factor for ICH^7^ - Smooth muscle cell degeneration^7^
COL4A1 mutation in mice	- Reasonable modelling of the clinical situation - Spontaneous ICH - Hypermuscularization, smooth muscle cell degeneration^6^ - BBB impairment^109^
CAA-related transgenic mice	- Reasonable modelling of the clinical situation - CAA^9^ - Microbleeds^9^
CCM/AVM-related transgenic mouse models	- Reasonable modelling of the clinical situation - Vascular malformations^15^^,^^16^^,^^18^
**(B) Non-rodent models**	

Autologous blood injection in rabbits	- Haematoma expansion^110^ - Haematoma evacuation^111^ - Perihaematomal oedema^111^ - BBB impairment^111^
Autologous blood injection in cats	- Perihaematomal oedema^112^ - Gyrencephalic brain - Higher white matter content
Autologous blood injection in dogs	- Haematoma evacuation^113^^,^^114^ - Gyrencephalic brain - Targeted white matter injury possible^113^ - BBB impairment^113^
Collagenase injection in dogs	- Haematoma expansion and evolution^115^ - Gyrencephalic brain - Higher white matter content
Vessel puncture in dogs	- Haematoma expansion^116^ - Cell death^116^ - Targeted lesion induction possible - Gyrencephalic brain - Higher white matter content
Autologous blood injection in pigs	- Endogenous haematoma clearance^117^, ^118^, ^119^ - White matter injury^33^ - Cell death^120^^,^^121^ - Perihaematomal oedema^33^^,^^122^ - Inflammation^33^ - Gyrencephalic brain - Higher white matter content
Autologous blood injection in sheep	- White matter injury^46^ - Cell death^46^ - Perihaematomal oedema^46^ - Gyrencephalic brain - Higher white matter content
Naturally occurring CAA in dogs	- CAA^123^ - Gyrencephalic brain - Higher white matter content - Clinically realistic model
CAA-related transgenic squirrel monkeys	- Reasonable modelling of the clinical situation - CAA^9^ - Microbleeds^9^

## Conclusion

**Figure 3:**
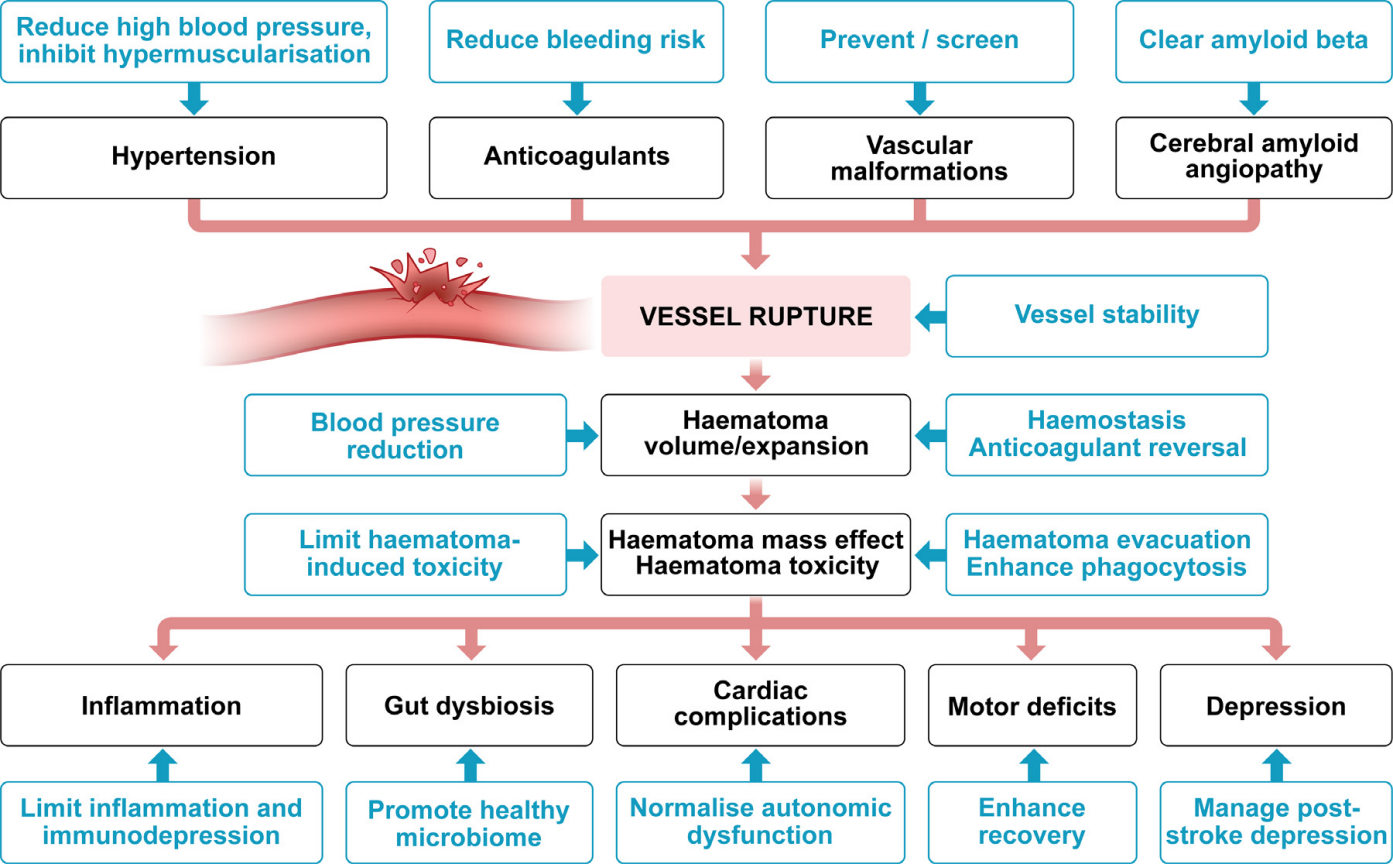
Therapeutic targets and approaches in ICH.

Over the past two decades, it has become clear that ICH was previously neglected as a stroke type being distinct in its pathophysiology and treatment needs. We here summarized current therapeutical approaches and unaddressed targets that will help researchers in clinical translation (Figure 3). Importantly, there is increasing knowledge about the involvement of other cell types and organs that need more thorough investigation and to be considered for successful interventions. This also means that animal modelling needs to reflect this situation more closely to target the aetiology and pathophysiology of ICH better.

## Outstanding questions

Whereas much has been learned about the pathological mechanisms involved in ICH-induced brain injury, translating that information into novel therapies remains a challenge. Some of the mechanisms elucidated may have beneficial as well as detrimental effects (e.g., inflammation), the impact of which may vary with haematoma size and time after ictus. In addition, the importance of different injury mechanisms may differ between human and animal models. The use of large gyrencephalic species in addition to rodents for ICH modelling may address some concerns, but even they do not perfectly replicate human ICH size and time course. Thus, it is imperative to select the ICH model best suited to the respective research question or therapeutic target for best translational value.

## Search strategy and selection criteria

Data for this review were identified by searches of MEDLINE, Current Contents, PubMed, and references from relevant articles using the search terms “intracerebral hemorrhage”, “hypertension”, “smooth muscle cells”, “hypercholesterolaemia”, “anticoagulants”, “haematoma expansion”, “haematoma evacuation”, “haematoma resolution”, “oedema”, “white matter injury”, “demyelination”, “axonal degeneration”, “ferroptosis”, “lipid peroxidation”, “brain endothelial cells”, “inflammation”, “small RNA”, “miRNA”, “exosomes”, “nanoparticles”, “cardiac complication”, “brain-heart axis”, “immunosuppression”, “gut-brain axis”, “recovery”, and “depression”. Only articles published in English were included with a particular focus on the past 3 years.

### Contributions

All authors contributed to the writing of the article and approved the final version of the manuscript.

## Declaration of interests

The authors declare that they have no conflicts of interests.

## Appendix Supplementary materials

Image, application 1

## References

[1] GBD Stroke Collaborators (2021). Global, regional, and national burden of stroke and its risk factors, 1990–2019: a systematic analysis for the global burden of disease study 2019. Lancet Neurol.

[2] Chin J.H., Vora N. (2014). The global burden of neurologic diseases. Neurology.

[3] Iida S., Baumbach G.L., Lavoie J.L. (2005). Spontaneous stroke in a genetic model of hypertension in mice. Stroke.

[4] Wakisaka Y., Chu Y., Miller J.D. (2010). Spontaneous intracerebral hemorrhage during acute and chronic hypertension in mice. J Cereb Blood Flow Metab.

[5] Bhatia P.M., Chamberlain R., Luo X. (2012). Elevated blood pressure causes larger hematoma in a rat model of intracerebral hemorrhage. Transl Stroke Res.

[6] Ratelade J., Klug N.R., Lombardi D. (2020). Reducing hypermuscularization of the transitional segment between arterioles and capillaries protects against spontaneous intracerebral hemorrhage. Circulation.

[7] Xiao N., Liu T.L., Li H. (2020). Low serum uric acid levels promote hypertensive intracerebral hemorrhage by disrupting the smooth muscle cell-elastin contractile unit and upregulating the Erk1/2-MMP axis. Transl Stroke Res.

[8] Pinho J., Araujo J.M., Costa A.S. (2021). Intracerebral hemorrhage recurrence in patients with and without cerebral amyloid angiopathy. Cerebrovasc Dis Extra.

[9] Alharbi B.M., Tso M.K., Macdonald R.L. (2016). Animal models of spontaneous intracerebral hemorrhage. Neurol Res.

[10] Inoue Y., Ando Y., Misumi Y. (2021). Current management and therapeutic strategies for cerebral amyloid angiopathy. Int J Mol Sci.

[11] Bai Y., Deng H., Shantsila A. (2017). Rivaroxaban versus dabigatran or warfarin in real-world studies of stroke prevention in atrial fibrillation: systematic review and meta-analysis. Stroke.

[12] Foerch C., Lo E.H., van Leyen K. (2019). Intracerebral hemorrhage formation under direct oral anticoagulants. Stroke.

[13] Na S.Y., Mracsko E., van Ryn J. (2015). Idarucizumab improves outcome in murine brain hemorrhage related to dabigatran. Ann Neurol.

[14] Petrault M., Ouk T., Petrault O. (2019). Safety of oral anticoagulants on experimental brain microbleeding and cognition. Neuropharmacology.

[15] Cardoso C., Arnould M., De Luca C. (2020). Novel chronic mouse model of cerebral cavernous malformations. Stroke.

[16] McDonald D.A., Shenkar R., Shi C. (2011). A novel mouse model of cerebral cavernous malformations based on the two-hit mutation hypothesis recapitulates the human disease. Hum Mol Genet.

[17] Nikolaev S.I., Vetiska S., Bonilla X. (2018). Somatic activating KRAS mutations in arteriovenous malformations of the brain. New Engl J Med.

[18] Li Q.F., Decker-Rockefeller B., Bajaj A. (2018). Activation of ras in the vascular endothelium induces brain vascular malformations and hemorrhagic stroke. Cell Rep.

[19] Rist P.M., Buring J.E., Ridker P.M. (2019). Lipid levels and the risk of hemorrhagic stroke among women. Neurology.

[20] Li X., Cheng X., Wang X. (2020). Dyslipidemic diet induces mobilization of peripheral neutrophils and monocytes that exacerbate hemorrhagic brain injury and neuroinflammation. Front Cell Neurosci.

[21] Sandset E.C., Anderson C.S., Bath P.M. (2021). European Stroke Organisation (ESO) guidelines on blood pressure management in acute ischaemic stroke and intracerebral haemorrhage. Eur Stroke J.

[22] Chu H., Gao Z., Huang C. (2020). Relationship between hematoma expansion induced by hypertension and hyperglycemia and blood-brain barrier disruption in mice and its possible mechanism: role of aquaporin-4 and connexin43. Neurosci Bull.

[23] Schlunk F., Böhm M., Boulouis G. (2019). Secondary bleeding during acute experimental intracerebral hemorrhage. Stroke.

[24] Hanley D.F., Thompson R.E., Rosenblum M. (2019). Efficacy and safety of minimally invasive surgery with thrombolysis in intracerebral haemorrhage evacuation (MISTIE III): a randomised, controlled, open-label, blinded endpoint phase 3 trial. Lancet.

[25] Mendelow A.D., Gregson B.A., Rowan E.N. (2013). Early surgery versus initial conservative treatment in patients with spontaneous supratentorial lobar intracerebral haematomas (STICH II): a randomised trial. Lancet.

[26] Chang C.F., Massey J., Osherov A. (2020). Bexarotene enhances macrophage erythrophagocytosis and hematoma clearance in experimental intracerebral hemorrhage. Stroke.

[27] Wang G., Li T., Duan S.N. (2018). PPAR-γ promotes hematoma clearance through haptoglobin-hemoglobin-CD163 in a rat model of intracerebral hemorrhage. Behav Neurol.

[28] Xu J., Chen Z., Yu F. (2020). IL-4/STAT6 signaling facilitates innate hematoma resolution and neurological recovery after hemorrhagic stroke in mice. Proc Natl Acad Sci USA.

[29] Jing C., Bian L., Wang M. (2019). Enhancement of hematoma clearance with CD47 blocking antibody in experimental intracerebral hemorrhage. Stroke.

[30] Fu X., Zhou G., Zhuang J. (2021). White matter injury after intracerebral hemorrhage. Front Neurol.

[31] Yang Y., Chen X., Feng Z. (2022). MEC17-induced alpha-tubulin acetylation restores mitochondrial transport function and alleviates axonal injury after intracerebral hemorrhage in mice. J Neurochem.

[32] Yang H., Ni W., Wei P. (2021). HDAC inhibition reduces white matter injury after intracerebral hemorrhage. J Cereb Blood Flow Metab.

[33] Yang H., Gao X.J., Li Y.J. (2019). Minocycline reduces intracerebral hemorrhage-induced white matter injury in piglets. CNS Neurosci Ther.

[34] Yang Z., Dong S., Zheng Q. (2019). FTY720 attenuates iron deposition and glial responses in improving delayed lesion and long-term outcomes of collagenase-induced intracerebral hemorrhage. Brain Res.

[35] Fouda A.Y., Newsome A.S., Spellicy S. (2017). Minocycline in acute cerebral hemorrhage: an early phase randomized trial. Stroke.

[36] Zille M., Karuppagounder S.S., Chen Y. (2017). Neuronal death after hemorrhagic stroke *in vitro* and *in vivo* shares features of ferroptosis and necroptosis. Stroke.

[37] Li Q., Weiland A., Chen X. (2018). Ultrastructural characteristics of neuronal death and white matter injury in mouse brain tissues after intracerebral hemorrhage: coexistence of ferroptosis, autophagy, and necrosis. Front Neurol.

[38] Karuppagounder S.S., Alin L., Chen Y. (2018). N-acetylcysteine targets 5 lipoxygenase-derived, toxic lipids and can synergize with prostaglandin E2 to inhibit ferroptosis and improve outcomes following hemorrhagic stroke in mice. Ann Neurol.

[39] Zhou S.Y., Cui G.Z., Yan X.L. (2020). Mechanism of ferroptosis and its relationships with other types of programmed cell death: insights for potential interventions after intracerebral hemorrhage. Front Neurosci.

[40] Li Q., Han X., Lan X. (2017). Inhibition of neuronal ferroptosis protects hemorrhagic brain. JCI Insight.

[41] Chen B., Chen Z., Liu M. (2019). Inhibition of neuronal ferroptosis in the acute phase of intracerebral hemorrhage shows long-term cerebroprotective effects. Brain Res Bull.

[42] Alim I., Caulfield J.T., Chen Y. (2019). Selenium drives a transcriptional adaptive program to block ferroptosis and treat stroke. Cell.

[43] Selim M., Foster L.D., Moy C.S. (2019). Deferoxamine mesylate in patients with intracerebral haemorrhage (i-DEF): a multicentre, randomised, placebo-controlled, double-blind phase 2 trial. Lancet Neurol.

[44] Zille M., Ikhsan M., Jiang Y. (2019). The impact of endothelial cell death in the brain and its role after stroke: a systematic review. Cell Stress.

[45] Jia P., He J., Li Z. (2021). Profiling of blood-brain barrier disruption in mouse intracerebral hemorrhage models: collagenase injection vs. autologous arterial whole blood infusion. Front Cell Neurosci.

[46] Boltze J., Ferrara F., Hainsworth A.H. (2019). Lesional and perilesional tissue characterization by automated image processing in a novel gyrencephalic animal model of peracute intracerebral hemorrhage. J Cereb Blood Flow Metab.

[47] Keep R.F., Andjelkovic A.V., Xiang J. (2018). Brain endothelial cell junctions after cerebral hemorrhage: changes, mechanisms and therapeutic targets. J Cereb Blood Flow Metab.

[48] Kung T.F.C., Wilkinson C.M., Dirks C.A. (2021). Glibenclamide does not improve outcome following severe collagenase-induced intracerebral hemorrhage in rats. PLoS One.

[49] Xu F., Shen G., Su Z. (2019). Glibenclamide ameliorates the disrupted blood-brain barrier in experimental intracerebral hemorrhage by inhibiting the activation of NLRP3 inflammasome. Brain Behav.

[50] Xue M., Yong V.W. (2020). Neuroinflammation in intracerebral haemorrhage: immunotherapies with potential for translation. Lancet Neurol.

[51] Bai Q., Xue M., Yong V.W. (2020). Microglia and macrophage phenotypes in intracerebral haemorrhage injury: therapeutic opportunities. Brain J Neurol.

[52] Tschoe C., Bushnell C.D., Duncan P.W. (2020). Neuroinflammation after intracerebral hemorrhage and potential therapeutic targets. J Stroke.

[53] Zhao X., Ting S.M., Liu C.H. (2017). Neutrophil polarization by IL-27 as a therapeutic target for intracerebral hemorrhage. Nat Commun.

[54] Zhao X., Kruzel M., Ting S.M. (2021). Optimized lactoferrin as a highly promising treatment for intracerebral hemorrhage: pre-clinical experience. J Cereb Blood Flow Metab.

[55] Zhang J., Shi K., Li Z. (2018). Organ- and cell-specific immune responses are associated with the outcomes of intracerebral hemorrhage. FASEB J.

[56] Goods B.A., Askenase M.H., Markarian E. (2021). Leukocyte dynamics after intracerebral hemorrhage in a living patient reveal rapid adaptations to tissue milieu. JCI Insight.

[57] Mei S., Shao Y., Fang Y. (2021). The changes of leukocytes in brain and blood after intracerebral hemorrhage. Front Immunol.

[58] Han R., Luo J., Shi Y. (2017). PD-L1 (programmed death ligand 1) protects against experimental intracerebral hemorrhage-induced brain injury. Stroke.

[59] Zhang L., Wuri J., An L. (2021). Metoprolol attenuates intracerebral hemorrhage-induced cardiac damage by suppression of sympathetic overactivity in mice. Auton Neurosci Basic Clin.

[60] Fu Y., Hao J., Zhang N. (2014). Fingolimod for the treatment of intracerebral hemorrhage: a 2-arm proof-of-concept study. JAMA Neurol.

[61] Yu X., Zhou G., Shao B. (2021). Gut microbiota dysbiosis induced by intracerebral hemorrhage aggravates neuroinflammation in mice. Front Microbiol.

[62] Rass V., Lindner A., Ianosi B.A. (2021). Early alterations in heart rate are associated with poor outcome in patients with intracerebral hemorrhage. J Crit Care.

[63] Li W., Li L., Chopp M. (2018). Intracerebral hemorrhage induces cardiac dysfunction in mice without primary cardiac disease. Front Neurol.

[64] Li W., Li L., Li W. (2020). Spleen associated immune-response mediates brain-heart interaction after intracerebral hemorrhage. Exp Neurol.

[65] Shi X., Bai H., Wang J. (2021). Behavioral assessment of sensory, motor, emotion, and cognition in rodent models of intracerebral hemorrhage. Front Neurol.

[66] Balkaya M.G., Trueman R.C., Boltze J. (2018). Behavioral outcome measures to improve experimental stroke research. Behav Brain Res.

[67] Tamakoshi K., Ishida K., Hayao K. (2018). Behavioral effect of short- and long-term exercise on motor functional recovery after intracerebral hemorrhage in rats. J Stroke Cerebrovasc Dis Off J Natl Stroke Assoc.

[68] Okuda Y., Nakata T. (2020). Effect of intensive rehabilitation on improvement of activity of daily living after intracerebral hemorrhage: a retrospective observational study. Int J Rehabil Res.

[69] MacLellan C.L., Plummer N., Silasi G. (2011). Rehabilitation promotes recovery after whole blood-induced intracerebral hemorrhage in rats. Neurorehabil Neural Repair.

[70] Langhorne P., Wu O., Rodgers H. (2017). A Very Early Rehabilitation Trial after stroke (AVERT): a phase III, multicentre, randomised controlled trial. Health Technol Assess.

[71] Liu Y., Lu G., Su X.W. (2018). Characterization of axon damage, neurological deficits, and histopathology in two experimental models of intracerebral hemorrhage. Front Neurosci.

[72] Kubiszewski P., Sugita L., Kourkoulis C. (2020). Association of selective serotonin reuptake inhibitor use after intracerebral hemorrhage with hemorrhage recurrence and depression severity. JAMA Neurol.

[73] Zhu W., Gao Y., Wan J. (2018). Changes in motor function, cognition, and emotion-related behavior after right hemispheric intracerebral hemorrhage in various brain regions of mouse. Brain Behav Immun.

[74] Singh N., Bansal Y., Bhandari R. (2017). Naringin reverses neurobehavioral and biochemical alterations in intracerebroventricular collagenase-induced intracerebral hemorrhage in rats. Pharmacology.

[75] MacLellan C.L., Langdon K.D., Churchill K.P. (2009). Assessing cognitive function after intracerebral hemorrhage in rats. Behav Brain Res.

[76] Zou C., Huang X., Lan X. (2021). Potential genes and mechanisms linking intracerebral hemorrhage and depression: a bioinformatics-based study. Int J Gen Med.

[77] Wu Y., Wang L., Hu K. (2018). Mechanisms and therapeutic targets of depression after intracerebral hemorrhage. Front Psychiatry.

[78] Martinez B., Peplow P.V. (2017). Blood microRNAs as potential diagnostic markers for hemorrhagic stroke. Neural Regen Res.

[79] Cheng X., Ander B.P., Jickling G.C. (2020). MicroRNA and their target mRNAs change expression in whole blood of patients after intracerebral hemorrhage. J Cereb Blood Flow Metab.

[80] Wan S.Y., Li G.S., Tu C. (2021). MicroNAR-194-5p hinders the activation of NLRP3 inflammasomes and alleviates neuroinflammation during intracerebral hemorrhage by blocking the interaction between TRAF6 and NLRP3. Brain Res.

[81] Hu L., Zhang H., Wang B. (2020). MicroRNA-152 attenuates neuroinflammation in intracerebral hemorrhage by inhibiting thioredoxin interacting protein (TXNIP)-mediated NLRP3 inflammasome activation. Int Immunopharmacol.

[82] Xi T., Jin F., Zhu Y. (2018). miR-27a-3p protects against blood-brain barrier disruption and brain injury after intracerebral hemorrhage by targeting endothelial aquaporin-11. J Biol Chem.

[83] Fu X., Niu T., Li X. (2019). MicroRNA-126-3p attenuates intracerebral hemorrhage-induced blood-brain barrier disruption by regulating VCAM-1 expression. Front Neurosci.

[84] Cheng H.Y., Wang Y.S., Hsu P.Y. (2019). miR-195 Has a Potential to Treat Ischemic and Hemorrhagic Stroke through Neurovascular Protection and Neurogenesis. Mol Ther Methods Clin Dev.

[85] Li P.F., Guo S.C., Liu T. (2020). Integrative analysis of transcriptomes highlights potential functions of transfer-RNA-derived small RNAs in experimental intracerebral hemorrhage. Aging (Albany NY).

[86] Nguyen T.T.M., van der Bent M.L., Wermer M.J.H. (2020). Circulating tRNA fragments as a novel biomarker class to distinguish acute stroke subtypes. Int J Mol Sci.

[87] Li M., Li X., Wang D. (2021). Inhibition of exosome release augments neuroinflammation following intracerebral hemorrhage. FASEB J.

[88] Walsh K.B., Campos B., Hart K. (2017). M2 monocyte microparticles are increased in intracerebral hemorrhage. J Stroke Cerebrovasc Dis.

[89] Duan S., Wang F., Cao J. (2020). Exosomes derived from MicroRNA-146a-5p-enriched bone marrow mesenchymal stem cells alleviate intracerebral hemorrhage by inhibiting neuronal apoptosis and microglial M1 polarization. Drug Des Dev Ther.

[90] Yi X., Tang X. (2021). Exosomes from miR-19b-3p-modified ADSCs inhibit ferroptosis in intracerebral hemorrhage mice. Front Cell Dev Biol.

[91] Shen H., Yao X., Li H. (2018). Role of exosomes derived from miR-133b modified MSCs in an experimental rat model of intracerebral hemorrhage. J Mol Neurosci.

[92] Kang D.W., Kim C.K., Jeong H.G. (2017). Biocompatible custom ceria nanoparticles against reactive oxygen species resolve acute inflammatory reaction after intracerebral hemorrhage. Nano Res.

[93] Mo Y., Duan L., Yang Y. (2021). Nanoparticles improved resveratrol brain delivery and its therapeutic efficacy against intracerebral hemorrhage. Nanoscale.

[94] Dharmalingam P., Talakatta G., Mitra J. (2020). Pervasive genomic damage in experimental intracerebral hemorrhage: therapeutic potential of a mechanistic-based carbon nanoparticle. ACS Nano.

[95] Dang L., Dong X., Yang J. (2021). Influence of nanoparticle-loaded edaravone on postoperative effects in patients with cerebral hemorrhage. J Nanosci Nanotechnol.

[96] Hemorrhagic Stroke Academia Industry Roundtable Participants (2018). Basic and translational research in intracerebral hemorrhage: limitations, priorities, and recommendations. Stroke.

[97] Gokhale S., Caplan L.R., James M.L. (2015). Sex differences in incidence, pathophysiology, and outcome of primary intracerebral hemorrhage. Stroke.

[98] Hsieh J.T., Ang B.T., Ng Y.P. (2016). Comparison of gender differences in intracerebral hemorrhage in a multi-ethnic Asian population. PLoS One.

[99] Fisher M., Feuerstein G., Howells D.W. (2009). Update of the stroke therapy academic industry roundtable preclinical recommendations. Stroke.

[100] Yang Y., Chen X., Feng Z. (2021). MEC17-induced alpha-tubulin acetylation restores mitochondrial transport function and alleviates axonal injury after intracerebral hemorrhage in mice. J Neurochem.

[101] Zhu W., Gao Y., Chang C.F. (2014). Mouse models of intracerebral hemorrhage in ventricle, cortex, and hippocampus by injections of autologous blood or collagenase. PLoS One.

[102] Zheng J., Shi L., Liang F. (2018). Sirt3 ameliorates oxidative stress and mitochondrial dysfunction after intracerebral hemorrhage in diabetic rats. Front Neurosci.

[103] Liesz A., Middelhoff M., Zhou W. (2011). Comparison of humoral neuroinflammation and adhesion molecule expression in two models of experimental intracerebral hemorrhage. Exp Transl Stroke Med.

[104] Bahader G.A., Nash K.M., Almarghalani D.A. (2021). Type-I diabetes aggravates post-hemorrhagic stroke cognitive impairment by augmenting oxidative stress and neuroinflammation in mice. Neurochem Int.

[105] Hijioka M., Anan J., Matsushita H. (2016). Axonal dysfunction in internal capsule is closely associated with early motor deficits after intracerebral hemorrhage in mice. Neurosci Res.

[106] Lakovic K., Ai J., D'Abbondanza J. (2014). Bilirubin and its oxidation products damage brain white matter. J Cereb Blood Flow Metab.

[107] Lauer A., Cianchetti F.A., Van Cott E.M. (2011). Anticoagulation with the oral direct thrombin inhibitor dabigatran does not enlarge hematoma volume in experimental intracerebral hemorrhage. Circulation.

[108] Meissner A., Minnerup J., Soria G. (2017). Structural and functional brain alterations in a murine model of Angiotensin II-induced hypertension. J Neurochem.

[109] Ratelade J., Mezouar N., Domenga-Denier V. (2018). Severity of arterial defects in the retina correlates with the burden of intracerebral haemorrhage in COL4A1-related stroke. J Pathol.

[110] Chen M., Yan Q., Sun J. (2017). Investigating the relationship between cerebrospinal fluid and magnetic induction phase shift in rabbit intracerebral hematoma expansion monitoring by MRI. Sci Rep.

[111] Wu G., Wu J., Jiao Y. (2015). Rosiglitazone infusion therapy following minimally invasive surgery for intracerebral hemorrhage evacuation decreases matrix metalloproteinase-9 and blood-brain barrier disruption in rabbits. BMC Neurol.

[112] Lin X., Tang Y., Sun B. (2010). Cerebral glucose metabolism: Influence on perihematomal edema formation after intracerebral hemorrhage in cat models. Acta Radiol.

[113] Wu G., Wang F., Wang L. (2017). Minimally invasive surgery for evacuating the intracerebral hematoma in early stages decreased secondary damages to the internal capsule in dog model of ICH observed by diffusion tensor imaging. J Stroke Cerebrovasc Dis.

[114] Wu G., Zhong W. (2010). Effect of minimally invasive surgery for cerebral hematoma evacuation in different stages on motor evoked potential and thrombin in dog model of intracranial hemorrhage. Neurol Res.

[115] An D., Park J., Shin J.I. (2015). Temporal Evolution of MRI characteristics in dogs with collagenase-induced intracerebral hemorrhage. Comp Med.

[116] Zhou X., Chen L., Feng C. (2013). Establishing an animal model of intracerebral hemorrhage under the guidance of ultrasound. Ultrasound Med Biol.

[117] Cao S., Zheng M., Hua Y. (2016). Hematoma changes during clot resolution after experimental intracerebral hemorrhage. Stroke.

[118] Hu S., Hua Y., Keep R.F. (2019). Deferoxamine therapy reduces brain hemin accumulation after intracerebral hemorrhage in piglets. Exp Neurol.

[119] Wang M., Xia F., Wan S. (2021). Role of complement component 3 in early erythrolysis in the hematoma after experimental intracerebral hemorrhage. Stroke.

[120] Gu Y., Hua Y., Keep R.F. (2009). Deferoxamine reduces intracerebral hematoma-induced iron accumulation and neuronal death in piglets. Stroke.

[121] Liu R., Cao S., Hua Y. (2017). CD163 expression in neurons after experimental intracerebral hemorrhage. Stroke.

[122] Xie Q., Gu Y., Hua Y. (2014). Deferoxamine attenuates white matter injury in a piglet intracerebral hemorrhage model. Stroke.

[123] Uchida K., Miyauchi Y., Nakayama H. (1990). Amyloid angiopathy with cerebral hemorrhage and senile plaque in aged dogs. Nihon Juigaku Zasshi Jpn J Vet Sci.

