# Supplementary material for: Novel targets, treatments, and advanced models for intracerebral haemorrhage
Source: eBioMedicine. 2022 Feb 12;76:103880. doi: 10.1016/j.ebiom.2022.103880 (PMC8850756; doi:10.1016/j.ebiom.2022.103880)
Supplement: Supplementary file 1 [file mmc1.pdf]

Supplementary Material for

Novel targets, treatments, and advanced models for intracerebral haemorrhage

Marietta Zille[1], Tracy D. Farr[2], Richard F. Keep[3], Christine Römer[4], Guohua Xi[3], Johannes Boltze[5]

[1] University of Vienna, Department of Pharmaceutical Sciences, Division of Pharmacology and Toxicology, UZA II, Althanstr. 14, 1090 Vienna, Austria
[2] University of Nottingham, School of Life Sciences, Physiology, Pharmacology, and Neuroscience Division, Medical School, Nottingham NG7 2UH, UK
[3] University of Michigan, Department of Neurosurgery, Ann Arbor, MI 48109-2200, USA
[4] Max Delbrück Center for Molecular Medicine in the Helmholtz Association, The Berlin Institute for Medical Systems Biology, 13125 Berlin, Germany
[5] The University of Warwick, School of Life Sciences, Gibbet Hill Campus, Coventry CV4 7AL, UK

Correspondence to:
Marietta Zille, University of Vienna, Department of Pharmaceutical Sciences, Division of Pharmacology and Toxicology, UZA II, Althanstr. 14, 1090 Vienna, Austria, email: marietta.zille@univie.ac.at

Or

Johannes Boltze, University of Warwick, School of Life Sciences, Gibbet Hill Road, Coventry CV4 7AL, United Kingdom, email: johannes.boltze@warwick.ac.uk

Supplementary:

Supplementary Table. Overview of interventional clinical trials for spontaneous ICH after 2010. GOSE, Glasgow Outcome Scale Extended; MMP, matrix metalloproteinase; mRS, modified Rankin Scale; NIHSS, National Institute Health Stroke Scale; OR, odds ratio; rt-PA, recombinant tissue plasminogen activator.

Supplementary Table. Overview of interventional clinical trials for spontaneous ICH after 2010. GOSE, Glasgow Outcome Scale Extended; MMP, matrix metalloproteinase; mRS, modified Rankin Scale; NIHSS, National Institute Health Stroke Scale; OR, odds ratio; rt-PA, recombinant tissue plasminogen activator.

Study title (as listed in clinicaltrials.gov)	Identifier	Phase	Intervention	Target	Outcome (control vs. intervention)
Rivaroxaban Versus Dabigatran or Warfarin in Real-World Studies of Stroke Prevention in Atrial Fibrillation	Systematic review and meta-analysis (17 studies)	N/A	Drug: rivaroxaban, dabigatran, warfarin	Antiplatelet/ anticoagulation	Rivaroxaban similar risk of stroke/thromboembolism vs. dabigatran (hazard ratio 1.02) and reduced vs. warfarin (hazard ratio 0.75), increased major bleeding risk vs. dabigatran (hazard ratio 1.38) but similar to warfarin (hazard ratio 0.99)[1]
Apixaban After Anticoagulation-associated Intracerebral Haemorrhage in Patients With Atrial Fibrillation (APACHE-AF)	NCT02565693	2	Drug: apixaban	Antiplatelet/ anticoagulation	No difference in non-fatal stroke or vascular death (24% vs. 26%) and serious adverse events (57% bs. 58%) over median follow-up of 1.9 years[2]
NOACs for Stroke Prevention in Patients With Atrial Fibrillation and Previous ICH (NASPAF-ICH)	NCT02998905	2	Drug: non-vitamin K antagonist oral anticoagulants vs. acetylsalicylic acid	Antiplatelet/ anticoagulation	Completed, pending results
Study of Antithrombotic Treatment After IntraCerebral Haemorrhage (STATICH)	NCT03186729	4	Drug: antithrombotic agent	Antiplatelet/ anticoagulation	Recruiting
Anticoagulation in ICH Survivors for Stroke Prevention and Recovery (ASPIRE)	NCT03907046	3	Drug: apixaban, acetylsalicylic acid	Antiplatelet/ anticoagulation	Recruiting
PREvention of STroke in Intracerebral haemorrhaGE Survivors With Atrial Fibrillation (PRESTIGE-AF)	NCT03996772	3	Drug: apixaban, dabigatran, edoxaban, rivaroxaban	Antiplatelet/ anticoagulation	Recruiting
Antiplatelet Secondary Prevention International Randomised Trial After INtracerebral haemorrhaGe (ASPIRING)-Pilot Phase	NCT04522102	3	Drug: start antiplatelet monotherapy	Antiplatelet/ anticoagulation	Recruiting
Avoiding Anticoagulation After IntraCerebral Haemorrhage (A3ICH)	NCT03243175	3	Drug: apixaban, device: left atrial appendage closure	Antiplatelet/ anticoagulation	Recruiting
REstart or STop Antithrombotic Randomised Trial in France (RESTART-Fr)	NCT02966119	3	Drug: clopidogrel or acetylsalicylic acid and/or Dypyridamole	Antiplatelet/ anticoagulation	Recruiting
Early-Start Antiplatelet Treatment After Neurosurgery in Patients With Spontaneous Intracerebral Hemorrhage	NCT04820972	N/A	Drug: antiplatelet agents	Antiplatelet/ anticoagulation	Recruiting
Improving Platelet Activity for Cerebral Hemorrhage Treatment - DDAVP Proof of Concept (IMPACT)	NCT00961532	2	Drug: desmopressin	Antiplatelet reversal, haematoma expansion	Improved platelet activity, modest changes in haematoma growth[3]
Desmopressin for Reversal of Antiplatelet Drugs in Stroke Due to Haemorrhage (DASH)	NCT03696121	2	Drug: desmopressin	Antiplatelet reversal,	Recruiting

Study title (as listed in clinicaltrials.gov)	Identifier	Phase	Intervention	Target	Outcome (control vs. intervention)
				haematoma expansion	
Antihypertensive Treatment of Acute Cerebral Hemorrhage-II (ATACH-II)	NCT01176565	3	Drug: intravenous nicardipine hydrochloride	Haematoma expansion	Reduced rate of patients with haematoma expansion (24% vs. 19%), no difference in functional outcome mRS 0-3 (62% vs. 61%), or mortality (7% vs. 7%) at 90 days[4]
The Second Intensive Blood Pressure Reduction in Acute Cerebral Haemorrhage Trial (INTERACT2)	NCT00716079	3	Other: blood pressure management policies (routinely available drugs for blood pressure lowering)	Haematoma expansion	Reduced haematoma growth by 4.5% volume, improved functional outcome mRS 0-3 (61% vs. 64%), no difference in mortality (12% vs. 12%)[5]
The Third, Intensive Care Bundle With Blood Pressure Reduction in Acute Cerebral Hemorrhage Trial (INTERACT3)	NCT03209258	4 phases / 3 steps	Other: care bundle of active management (intensive blood pressure lowering, glycemic control, treatment of pyrexia, reversal of anticoagulation)	Haematoma expansion	Recruiting
Intensive Ambulance-delivered Blood Pressure Reduction in Hyper-Acute Stroke Trial (INTERACT4)	NCT03790800	3	Drug: urapidil	Haematoma expansion	Recruiting
Intracerebral Hemorrhage Acutely Decreasing Arterial Pressure Trial (ICH ADAPT)	NCT00963976	2	Drug: labetalol/ hydralazine/ enalapril	Haematoma expansion	No difference in haematoma growth (0.71 vs. 0.67), 90-day mRS (4 vs 2.5), or 30-day mortality (11% vs. 18%)[6]
The Intracerebral Hemorrhage Acutely Decreasing Arterial Pressure Trial II (ICH-ADAPT II)	NCT02281838	2	Drug: labetalol/ hydralazine/ enalapril	Haematoma expansion	Recruiting
Clevidipine in the Treatment of Patients With Acute Hypertension and Intracerebral Hemorrhage (ACCELERATE)	NCT00666328	3	Drug: clevidipine	Haematoma expansion	Achieved systolic blood pressure reduction, small haematoma volume change (-2.9%)[7]
Esmolol for the Treatment of Hypertension After Intracerebral Hemorrhage Study (ETHICHS)	NCT03743103	4	Drug: brevibloc (esmolol hydrochloride), nitroprusside	Haematoma expansion	Recruiting
Triple Therapy Prevention of Recurrent Intracerebral Disease EveNts Trial (TRIDENT)	NCT02699645	3	Drug: telmisartan, amlodipine, and indapamide	Haematoma expansion	Recruiting
Regulating Blood Pressure During Recovery From Intracerebral Hemorrhage (REDUCE)	NCT04760717	2	Drug: Spironolactone	Haematoma expansion	Recruiting
Analgesia-first Minimal Sedation for Spontaneous Intracerebral Hemorrhage Early Antihypertensive Treatment (ASSICHH)	NCT03207100	N/A	Combination Product: Analgesia-first minimal sedation and antihypertensive treatment	Haematoma expansion	Completed, pending results
Tranexamic Acid for Acute ICH Growth 3reatment by Spot Sign (TRAIGE)	NCT02625948	2	Drug: tranexamic acid	Haematoma expansion	Recruiting

**Study title (as listed in clinicaltrials.gov)**	**Identifier**	**Phase**	**Intervention**	**Target**	**Outcome (control vs. intervention)**
Treatment of Intracerebral Hemorrhage in Patients on Non-vitamin K Antagonist (TICH-NOAC)	NCT02866838	2/3	Drug: tranexamic acid	Haematoma expansion	Completed, pending results
Tranexamic acid for hyperacute primary IntraCerebral Haemorrhage (TICH-2)	ISRCTN93732214	3	Drug: tranexamic acid	Haematoma expansion	Reduced rate of patients with haematoma expansion (29% vs. 25%), no difference in favourable functional outcome mRS 0-3 (46% vs. 45%) and mortality (21% vs. 22%) at 90 days[8]
Tranexamic Acid for Spontaneous Acute Cerebral Hemorrhage Trial (TRANSACT)	NCT03044184	3	Drug: tranexamic acid	Haematoma expansion	Recruiting
The Spot Sign and Tranexamic Acid On Preventing ICH Growth – AUStralasia Trial (STOP-AUST)	NCT01702636	2	Drug: tranexamic acid	Haematoma expansion	No difference in the rate of patients with haematoma expansion (52% vs. 44%), favourable functional outcome mRS 0-3 (46% vs. 56%) or death (16% vs. 26%)[9]
Stopping Haemorrhage With Tranexamic Acid for Hyperacute Onset Presentation Including Mobile Stroke Units (STOP-MSU)	NCT03385928	2	Drug: tranexamic acid	Haematoma expansion	Recruiting
Effectiveness of Intravenous Tranexamic Acid in Primary Cerebral Hemorrhage for Prevention of Hematoma Progression	NCT04742205	1	Biological: recombinant activated factor VII	Haematoma expansion	Not yet recruiting
Factor VIIa in Acute Intracerebral Haemorrhage	NCT00266006	2	Drug: eptacog alfa (activated)	Haematoma expansion	Completed, pending results
Recombinant Factor VIIa (rFVIIa) for Hemorrhagic Stroke Trial (FASTEST)	NCT03496883	3	Biological: recombinant activated factor VII	Haematoma expansion	Recruiting
Safety and Preliminary Efficacy of Activated Recombinant Human Factor VII for Preventing Early Hematoma Growth in Acute Intracerebral Haemorrhage	NCT01563445	2	Biological: activated recombinant human factor VII	Haematoma expansion	Completed, pending results
Safety and Preliminary Efficacy of Activated Recombinant Human Factor VII in Acute Intracerebral Haemorrhage	NCT01566786	2	Biological: recombinant activated factor VII	Haematoma expansion	Completed, pending results
"Spot Sign" Selection of Intracerebral Hemorrhage to Guide Hemostatic Therapy (SPOTLIGHT)	NCT01359202	2	Biological: recombinant activated factor VII	Haematoma expansion	No difference in haematoma growth (2.6 vs. 2.5 mL) at 24 hours, favourable functional outcome mRS 0-3 (32% vs. 37%), or mortality (21% vs. 20%) at 90 days[10]
The Spot Sign for Predicting and Treating ICH Growth Study (STOP-IT)	NCT00810888	2	Biological: recombinant activated factor VII	Haematoma expansion	
Clinical Study on the Treatment of Hypertensive Intracerebral Hemorrhage With Panax Notoginseng Saponin (CSTHIHPNS)	NCT02999048	4	Drug: Panax notoginseng saponins	Haematoma expansion	Completed, pending results
Surgical trial in lobar intracerebral hemorrhage (STICH II)	ISRCTN22153967	N/A	Surgical: craniotomy	Haematoma evacuation	No difference in mortality (24% vs. 18%) or favourable functional outcome GOSE (38% vs. 41%) at 6-months[11]

Study title (as listed in clinicaltrials.gov)	Identifier	Phase	Intervention	Target	Outcome (control vs. intervention)
Decompressive Hemicraniectomy in Intracerebral Hemorrhage (SWITCH)	NCT02258919	N/A	Surgical: decompressive craniectomy	Haematoma evacuation	Recruiting
Minimally invasive stereotactic puncture and thrombolysis therapy versus conventional craniotomy in the treatment of acute intracerebral hemorrhage	ACTRN126100009 45022	N/A	Procedure: minimally invasive stereotactic puncture and thrombolysis therapy	Haematoma evacuation	Improved functional outcome (mRS 3.9 vs. 2.2), no difference in mortality (24% vs. 19%) at 365 days[12]
The Minimally Invasive Endoscopic Surgery With the Axonpen System for Spontaneous Intracerebral Hemorrhage (MIECH)	NCT04839770	N/A	Device: Axonpen system	Haematoma evacuation	Recruiting
Minimally invasive craniopuncture combined with urokinase infusion therapy	N/A	N/A	Surgical: minimally invasive craniopuncture therapy, Drug: urokinase	Haematoma evacuation	No difference in functional outcome mRS 0-3 (19% vs. 14%) at 14 days, decreased mortality (25% vs. 15%) at 90 days[13]
Minimally Invasive Surgery and rtPA for Intracerebral Hemorrhage Evacuation (MISTIE)	NCT00224770	2	Surgical: minimally invasive surgery, Biological: rt-PA	Haematoma evacuation	No difference in favourable outcome mRS 0-3 (19% vs. 32%) and mortality (35.5% vs. 40%) at 365 days[14]
Minimally invasive surgery plus rt-PA for ICH evacuation phase III (MISTIE-III)	NCT01827046	3	Surgical: minimally invasive surgery, Biological: rt-PA	Haematoma evacuation	No difference in favourable functional outcome mRS 0-3 at 12-months for moderate to large ICH (42% vs. 44%), reduced mortality (26% vs. 19%),[15] patients with ≤15 mL end of treatment ICH volume or ≥70% volume reduction with favourable functional outcomes[16]
Minimally-invasive Surgery Versus Craniotomy in Patients With Supratentorial Hypertensive Intracerebral Hemorrhage (MISICH)	NCT02811614	N/A	Surgical: minimally invasive surgery (endoscopic evacuation or stereotactic aspiration)	Haematoma evacuation	Recruiting
Early MiNimally-invasive Removal of IntraCerebral Hemorrhage (ICH) (ENRICH)	NCT02880878	N/A	Surgical: minimally invasive parafascicular surgery	Haematoma evacuation	Recruiting
Dutch Intracerebral Hemorrhage Surgery Trial Pilot Study (DIST pilot)	NCT03608423	2	Surgical: minimally-invasive endoscopy-guided surgery	Haematoma evacuation	Active, not recruiting
Early Minimally Invasive Image Guided Endoscopic Evacuation of Intracerebral Haemorrhage	NCT04805177	Pilot	Surgical: minimally invasive image-guided endoscopic hematoma evacuation	Haematoma evacuation	Recruiting
INVEST Feasibility – Minimally Invasive Endoscopic Surgery With Apollo in Patients With Brain Hemorrhage	NCT02654015		Surgical: Apollo minimally invasive surgery	Haematoma evacuation	Recruiting
Artemis in the Removal of Intracerebral Hemorrhage (MIND)	NCT03342664		Surgical: Artemis minimally invasive surgery	Haematoma evacuation	Recruiting
The MIRROR Registry: Minimally Invasive IntRaceRebral HemORrhage Evacuation (MIRROR)	NCT04494295		Surgical: Aurora Surgiscope System minimally invasive surgery	Haematoma evacuation	Recruiting

Study title (as listed in clinicaltrials.gov)	Identifier	Phase	Intervention	Target	Outcome (control vs. intervention)
Ultra-Early, Minimally inVAsive intraCerebral Haemorrhage evacUATion Versus Standard trEatment (EVACUATE)	NCT04434807	N/A	Surgical: minimally invasive hematoma evacuation	Haematoma evacuation	Recruiting
Minimal Invasive Surgical Intracerebral Hemorrhage Removal (HEALME)	NCT05138341	N/A	Surgical: minimally invasive hematoma evacuation	Haematoma evacuation	Not yet recruiting
Safety and Efficacy of Stereotactic Aspiration Plus Urokinase in Deep Intracerebral Hemorrhage Evacuation (STAPLE-dICH)	NCT04686877	2/3	Procedure: stereotactic aspiration plus urokinase irrigation (STAPLE)	Haematoma evacuation	Recruiting
Robotic Assisted Evacuation of Sub-acute and Chronic Hypertensive Cerebral Hemorrhage	NCT04957862	N/A	Procedure: robotic assisted evacuation	Haematoma evacuation	Not yet recruiting
Active Removal of IntraCerebral Hematoma Via Active Irrigation (ARCH)	NCT05118997	N/A	Procedure: IRRAflow with manual rt-PA administration followed by Active Fluid Exchange	Haematoma evacuation	Recruiting
Acute Hypertensive Cerebral Hemorrhage Surgery (NET-OCEAN)	NCT04957849	N/A	Procedure: neuroendoscopic, trans-occipital approach combined with low-drainage surgery	Haematoma evacuation	Not yet recruiting
Sterotactic Operation Integrating With Thrombolysis in Basal Ganglion Hemorrhage Evacuation (SOITBE)	NCT03957707	N/A	Surgical: minimally invasive puncture aspiration plus rt-PA	Haematoma evacuation	Recruiting
Stereotactic Operation Integrating With Thrombolysis in Basal Ganglion Hemorrhage Evacuation II (SOITBE II)	NCT04172376	N/A	Surgical: minimally invasive puncture aspiration plus rt-PA	Haematoma evacuation	Not yet recruiting
Intracavitary Injection of hUMSCs in Acute Basal Ganglia Hematoma After Stereotactic Aspiration	NCT04074408	2	Surgical: stereotactic aspiration surgery, Biological: human umbilical cord mesenchymal stem cells	Haematoma evacuation, stem cell transplantation	Recruiting
Use of Minocycline in Intracerebral Hemorrhage	NCT03040128	1/2	Drug: minocycline	White matter injury, inflammation	Decreased MMP-9 levels, no difference in 90-days mRS (1 vs. 1)[17]
A Pilot Study of Minocycline in Intracerebral Hemorrhage Patients (MACH)	NCT01805895	1/2	Drug: minocycline	White matter injury, inflammation	No difference in MMP-9 and interleukin 6 levels, no difference in 90-days mRS (3.7 vs. 3.5)[18]
Fingolimod as a Treatment of Cerebral Edema After Intracerebral Hemorrhage (FITCH)	NCT04088630	1	Drug: fingolimod	White matter injury, immunosuppression	Recruiting
Prevention of Hypertensive Injury to the Brain by Intensive Treatment in IntraCerebral Haemorrhage (PROHIBIT-ICH)	NCT03863665		Device: telemetric blood pressure monitoring	White matter injury, post-ICH cognitive impairment	Recruiting

Study title (as listed in clinicaltrials.gov)	Identifier	Phase	Intervention	Target	Outcome (control vs. intervention)
Intracerebral Hemorrhage Deferoxamine Trial (iDEF)	NCT02175225	2	Drug: deferoxamine mesylate	Iron toxicity, ferroptosis	Improved mRS 0-2 (36 vs. 45) and mRS 0-3 (68% vs. 72%), no difference in mortality (9% vs. 9%) at 180 days,[19] favourable outcome (mRS 0-2) for moderate hematoma volume (10-30 mL, 26% vs. 50%) at 90 days[20]
Clinical Study of Huperzine A in the Treatment of Patients With Hypertensive Cerebral Hemorrhage	NCT04509323	4	Drug: huperzine A	Cell death	Not yet recruiting
Edaravone Dexborneol for Treatment of Hypertensive Intracerebral Hemorrhage (ED-ICH)	NCT04714177	2	Drug: edaravone dexborneol	Cell death, neuroinflammation	Not yet recruiting
Feasibility Study of Transcranial Ultrasound Stimulation (TUS) on Stroke Patients	NCT04877184	N/A	Procedure: transcranial ultrasound stimulation and rehabilitation	Cell death, neuroinflammation, blood-brain barrier	Recruiting
Feasibility of Improving Cerebral Autoregulation in Acute Intracerebral Haemorrhage (BREATHE-ICH)	NCT03324321	N/A	Other: hypocapnia via hyperventilation protocol	Cerebral autoregulation	Improved autoregulatory index (4.8 pre-, 7.0 post-intervention)[21]
Glibenclamide Advantage in Treating Edema After Intracerebral Hemorrhage (GATE-ICH)	NCT03741530	N/A	Drug: glibenclamide	Perihaematomal oedema	Completed, pending results
Dimethyl Fumarate for the Treatment of Intracerebral Hemorrhage	NCT04890379	2	Drug: dimethyl fumarate	Perihaematomal oedema	Not yet recruiting
Conivaptan for the Reduction of Cerebral Edema in Intracerebral Hemorrhage- A Safety and Tolerability Study	NCT03000283	1	Drug: Conivaptan	Perihaematomal oedema	Safe and tolerable[22]
Administration of Celecoxib for Treatment of Intracerebral Hemorrhage: A Pilot Study (ACE-ICH)	NCT00526214	Pilot	Drug: celecoxib medication	Perihaematomal oedema, hematoma expansion	Reduced expansion of perihaematomal oedema (91% vs. 44%) and hematoma (49% vs. 2%), improved functional outcome mRS 0-2 (38% vs. 60%)[23]
Remote Ischemic Conditioning for Intracerebral Hemorrhage (RICH)	NCT03930940	1	Procedure: remote ischaemic conditioning	Perihaematomal oedema, hematoma resolution	Increased haematoma resolution (42% vs. 49%) and relative perihaematomalo edema (2.02 vs. 1.77) at 7 days, no difference in favourable outcome mRS 0-3 (60% vs. 65%)[24]
Remote Ischemic Conditioning for the Treatment of Intracerebral Hemorrhage (RICH-2)	NCT04657133	3	Procedure: remote ischaemic conditioning	Perihaematomal oedema, hematoma resolution	Recruiting
Remote Ischemic Conditioning for Cerebral Amyloid Angiopathy-related Intracerebral Hemorrhage (RIC-CAAH)	NCT04757597	N/A	Procedure: remote ischaemic conditioning	Perihaematomal oedema, hematoma resolution	Recruiting
Remote Ischemic Conditioning in Patients With Acute Stroke (RESIST)	NCT03481777	N/A	Procedure: remote ischaemic conditioning	Perihaematomal oedema, hematoma resolution	Recruiting

**Study title (as listed in clinicaltrials.gov)**	**Identifier**	**Phase**	**Intervention**	**Target**	**Outcome (control vs. intervention)**
Safety and Efficacy of Remote Ischemic Conditioning in Patients With Spontaneous Intracerebral Hemorrhage (SERIC-sICH)	NCT03484936	N/A	Procedure: remote ischaemic conditioning	Perihaematomal oedema, haematoma resolution	Not yet recruiting
Statin for Neuroprotection in Spontaneous Intracerebral Hemorrhage (STATIC)	NCT04857632	2/3	Drug: statin	Perihaematomal oedema, neuroinflammation	Recruiting
Statins In Intracerbral Hemorrhage (SATURN)	NCT03936361	3	Drug: statin	Perihaematomal oedema, neuroinflammation	Recruiting
Systemic Normothermia in Intracerebral Hemorrhage (ICH) (SNICH)	NCT02078037	1	Device: Arctic Sun cooling device	Perihaematomal oedema, neuroinflammation	Completed, pending results
Biomarker and Edema Attenuation in IntraCerebral Hemorrhage (BEACH)	NCT05020535	2	Drug: MW01-6-189WH	Perihaematomal oedema, neuroinflammation	Not yet recruiting
Chinese Herbal Medicine in Acute INtracerebral Haemorrhage (CHAIN)	NCT05066620	3	Drug: Chinese herbal medicine FYTF-919	Perihaematomal oedema, neuroinflammation	Not yet recruiting
Studying Anakinra to Reduce Secondary Brain Damage After Spontaneous Haemorrhagic Stroke (ACTION)	NCT04834388	2	Drug: interleukin 1 receptor antagonist Anakinra	Perihaematomal oedema, neuroinflammation, blood-brain barrier	Not yet recruiting
Interleukin-1 Receptor Antagonist in Intracerebral Haemorrhage (BLOC-ICH)	NCT03737344	2	Drug: interleukin 1 receptor antagonist Kineret	Perihaematomal oedema, neuroinflammation, blood-brain barrier	Completed, pending results
Safety of Pioglitazone for Hematoma Resolution In Intracerebral Hemorrhage (SHRINC)	NCT00827892	2	Drug: pioglitazone	Microglia, hematoma resolution	Completed, pending results
Efficacy, Safety and Tolerability of BAF312 Compared to Placebo in Patients With Intracerebral Hemorrhage (ICH)	NCT03338998	2	Drug: BAF312 (Siponimod, a selective modulator of sphingosine 1-phosphate receptor 1 and 5)	Neuroinflammation	Completed, pending results
A Proof of Concept Study to Evaluate CN-105 in ICH Patients (CATCH)	NCT03168581	2	Drug: CN-105	Neuroinflammation	Improved functional recovery mRS 0-3 (18% vs. 40%) at 30 days[25]
Evaluation of CN-105 in Subject With Acute Supratentorial Intracerebral Hemorrhage (S-CATCH)	NCT03711903	2	Drug: CN-105	Neuroinflammation	Recruiting

Study title (as listed in clinicaltrials.gov)	Identifier	Phase	Intervention	Target	Outcome (control vs. intervention)
Mesenchymal Stem Cells Therapy in Patients With Recent Intracerebral Hemorrhage	NCT03371329	1	Biological: mesenchymal stem cells	Neuroinflammation, regeneration	Completed, pending results
Clinical Evaluation of Removing Blood Stasis Therapy in Treating Acute Cerebral Hemorrhage Safety and Efficacy (CERBSTTSCH)	NCT03354026	4	Drug: traditional Chinese medicine (AICH-PXZY)	Gut microbiota	Not yet recruiting
Life After STroke - the LAST Study (LAST)	NCT01467206	N/A	Behavioural: Long-term follow up by a coordinating physiotherapist	Post-ICH motor recovery	No benefit on motor assessment or mRS (1.33 vs. 1.28) at 18 months[26]
A Very Early Rehabilitation Trial (AVERT)	ACTRN12606000 185561<br>NCT01846247	3	Procedure: very early mobilization vs. early mobilization	Post-ICH motor recovery	Poor functional outcome for very early mobilization (OR 0.48 favours early mobilization) and death (OR 3.21 favours early mobilization) at 3 months[27]
Akershus Early Mobilisation in Stroke Study (AKEMIS)	NCT00832351	3	Procedure: mobilization	Post-ICH motor recovery	Completed, pending results
Clinical Curative Effect and safe Research on treatment of hemorrhagic stroke with stage acupuncture	ChiCTR-TRC-08000225	N/A	Procedure: acupuncture	Post-ICH motor recovery	Improved lower limb function Fugl-Mayer (7 vs. 13 points difference from baseline), but not lower limb function (20 vs. 20 points difference) at 90 days[28]
Can Acupuncture Benefit Surgical Patients With Haemorrhagic Stroke?	NCT01037894	1/2	Procedure: acupuncture	Post-ICH motor recovery	Completed, pending results
Biologic Mechanisms of Early Exercise After Intracerebral Hemorrhage (BEACH)	NCT04027049	N/A	Device: Supine cycle ergometry of the lower extremities	Post-ICH motor recovery	Recruiting
Seizures Post Intracerebral Hemorrhage	NCT01115959	4	Drug: valproic acid	Post-ICH seizures	Completed, pending results
Effects of fluoxetine on functional outcomes after acute stroke (FOCUS)	ISRCTN83290762	N/A	Drug: fluoxetine	Post-ICH depression	No subgroup analysis for the haemorrhagic stroke patients only[29]
Assessment oF FluoxetINe In sTroke recoverY (AFFINITY)	ACTRN12611000 774921	3	Drug: fluoxetine	Post-ICH depression	No difference in shifts in mRS (OR 0.905)[30]
Safety and efficacy of fluoxetine on functional recovery after acute stroke (EFFECTS)	NCT02683213	N/A	Drug: fluoxetine	Post-ICH depression	No subgroup analysis for the haemorrhagic stroke patients only[31]

References

1. Bai Y, Deng H, Shantsila A, Lip GY. Rivaroxaban Versus Dabigatran or Warfarin in Real-World Studies of Stroke Prevention in Atrial Fibrillation: Systematic Review and Meta-Analysis. Stroke. 2017;48(4):970-6.
2. Schreuder F, van Nieuwenhuizen KM, Hofmeijer J, Vermeer SE, Kerkhoff H, Zock E, et al. Apixaban versus no anticoagulation after anticoagulation-associated intracerebral haemorrhage in patients with atrial fibrillation in the Netherlands (APACHE-AF): a randomised, open-label, phase 2 trial. The Lancet Neurology. 2021;20(11):907-16.
3. Naidech AM, Maas MB, Levasseur-Franklin KE, Liotta EM, Guth JC, Berman M, et al. Desmopressin improves platelet activity in acute intracerebral hemorrhage. Stroke. 2014;45(8):2451-3.
4. Qureshi AI, Palesch YY, Barsan WG, Hanley DF, Hsu CY, Martin RL, et al. Intensive Blood-Pressure Lowering in Patients with Acute Cerebral Hemorrhage. The New England journal of medicine. 2016;375(11):1033-43.
5. Anderson CS, Heeley E, Huang Y, Wang J, Stapf C, Delcourt C, et al. Rapid blood-pressure lowering in patients with acute intracerebral hemorrhage. The New England journal of medicine. 2013;368(25):2355-65.
6. Butcher KS, Jeerakathil T, Hill M, Demchuk AM, Dowlatshahi D, Coutts SB, et al. The Intracerebral Hemorrhage Acutely Decreasing Arterial Pressure Trial. Stroke. 2013;44(3):620-6.
7. Graffagnino C, Bergese S, Love J, Schneider D, Lazaridis C, LaPointe M, et al. Clevidipine rapidly and safely reduces blood pressure in acute intracerebral hemorrhage: the ACCELERATE trial. Cerebrovascular diseases. 2013;36(3):173-80.
8. Sprigg N, Flaherty K, Appleton JP, Al-Shahi Salman R, Bereczki D, Beridze M, et al. Tranexamic acid for hyperacute primary IntraCerebral Haemorrhage (TICH-2): an international randomised, placebo-controlled, phase 3 superiority trial. Lancet. 2018;391(10135):2107-15.
9. Meretoja A, Yassi N, Wu TY, Churilov L, Sibolt G, Jeng JS, et al. Tranexamic acid in patients with intracerebral haemorrhage (STOP-AUST): a multicentre, randomised, placebo-controlled, phase 2 trial. The Lancet Neurology. 2020;19(12):980-7.
10. Gladstone DJ, Aviv RI, Demchuk AM, Hill MD, Thorpe KE, Khoury JC, et al. Effect of Recombinant Activated Coagulation Factor VII on Hemorrhage Expansion Among Patients With Spot Sign-Positive Acute Intracerebral Hemorrhage: The SPOTLIGHT and STOP-IT Randomized Clinical Trials. JAMA neurology. 2019;76(12):1493-501.
11. Mendelow AD, Gregson BA, Rowan EN, Murray GD, Gholkar A, Mitchell PM, et al. Early surgery versus initial conservative treatment in patients with spontaneous supratentorial lobar intracerebral haematomas (STICH II): a randomised trial. Lancet. 2013;382(9890):397-408.
12. Zhou H, Zhang Y, Liu L, Han X, Tao Y, Tang Y, et al. A prospective controlled study: minimally invasive stereotactic puncture therapy versus conventional craniotomy in the treatment of acute intracerebral hemorrhage. BMC neurology. 2011;11:76.
13. Sun H, Liu H, Li D, Liu L, Yang J, Wang W. An effective treatment for cerebral hemorrhage: minimally invasive craniopuncture combined with urokinase infusion therapy. Neurological research. 2010;32(4):371-7.
14. Hanley DF, Thompson RE, Muschelli J, Rosenblum M, McBee N, Lane K, et al. Safety and efficacy of minimally invasive surgery plus alteplase in intracerebral haemorrhage evacuation (MISTIE): a randomised, controlled, open-label, phase 2 trial. The Lancet Neurology. 2016;15(12):1228-37.
15. Hanley DF, Thompson RE, Rosenblum M, Yenokyan G, Lane K, McBee N, et al. Efficacy and safety of minimally invasive surgery with thrombolysis in intracerebral haemorrhage evacuation (MISTIE III): a randomised, controlled, open-label, blinded endpoint phase 3 trial. Lancet. 2019;393(10175):1021-32.
16. Awad IA, Polster SP, Carrion-Penagos J, Thompson RE, Cao Y, Stadnik A, et al. Surgical Performance Determines Functional Outcome Benefit in the Minimally Invasive Surgery Plus Recombinant Tissue Plasminogen Activator for Intracerebral Hemorrhage Evacuation (MISTIE) Procedure. Neurosurgery. 2019;84(6):1157-68.
17. Chang JJ, Kim-Tenser M, Emanuel BA, Jones GM, Chapple K, Alikhani A, et al. Minocycline and matrix metalloproteinase inhibition in acute intracerebral hemorrhage: a pilot study. European journal of neurology. 2017;24(11):1384-91.
18. Fouda AY, Newsome AS, Spellicy S, Waller JL, Zhi W, Hess DC, et al. Minocycline in Acute Cerebral Hemorrhage: An Early Phase Randomized Trial. Stroke. 2017;48(10):2885-7.
19. Selim M, Foster LD, Moy CS, Xi G, Hill MD, Morgenstern LB, et al. Deferoxamine mesylate in patients with intracerebral haemorrhage (i-DEF): a multicentre, randomised, placebo-controlled, double-blind phase 2 trial. The Lancet Neurology. 2019;18(5):428-38.
20. Wei C, Wang J, Foster LD, Yeatts SD, Moy C, Mocco J, et al. Effect of Deferoxamine on Outcome According to Baseline Hematoma Volume: A Post Hoc Analysis of the i-DEF Trial. Stroke. 2021:STROKEAHA121035421.

21. Minhas JS, Panerai RB, Swienton D, Robinson TG. Feasibility of improving cerebral autoregulation in acute intracerebral hemorrhage (BREATHE-ICH) study: Results from an experimental interventional study. International journal of stroke : official journal of the International Stroke Society. 2020;15(6):627-37.
22. Corry JJ, Asaithambi G, Shaik AM, Lassig JP, Marino EH, Ho BM, et al. Conivaptan for the Reduction of Cerebral Edema in Intracerebral Hemorrhage: A Safety and Tolerability Study. Clinical drug investigation. 2020;40(5):503-9.
23. Lee SH, Park HK, Ryu WS, Lee JS, Bae HJ, Han MK, et al. Effects of celecoxib on hematoma and edema volumes in primary intracerebral hemorrhage: a multicenter randomized controlled trial. European journal of neurology. 2013;20(8):1161-9.
24. Zhao W, Jiang F, Li S, Liu G, Wu C, Wang Y, et al. Safety and efficacy of remote ischemic conditioning for the treatment of intracerebral hemorrhage: A proof-of-concept randomized controlled trial. International journal of stroke : official journal of the International Stroke Society. 2021:17474930211006580.
25. James ML, Troy J, Nowacki N, Komisarow J, Swisher CB, Tucker K, et al. CN-105 in Participants with Acute Supratentorial Intracerebral Hemorrhage (CATCH) Trial. Neurocritical care. 2021.
26. Askim T, Langhammer B, Ihle-Hansen H, Gunnes M, Lydersen S, Indredavik B, et al. Efficacy and Safety of Individualized Coaching After Stroke: the LAST Study (Life After Stroke): A Pragmatic Randomized Controlled Trial. Stroke. 2018;49(2):426-32.
27. Langhorne P, Wu O, Rodgers H, Ashburn A, Bernhardt J. A Very Early Rehabilitation Trial after stroke (AVERT): a Phase III, multicentre, randomised controlled trial. Health technology assessment. 2017;21(54):1-120.
28. Wang HQ, Hou M, Li H, Bao CL, Min L, Dong GR, et al. Effects of acupuncture treatment on motor function in patients with subacute hemorrhagic stroke: A randomized controlled study. Complementary therapies in medicine. 2020;49:102296.
29. Collaboration FT. Effects of fluoxetine on functional outcomes after acute stroke (FOCUS): a pragmatic, double-blind, randomised, controlled trial. Lancet. 2019;393(10168):265-74.
30. Collaboration AT. Safety and efficacy of fluoxetine on functional outcome after acute stroke (AFFINITY): a randomised, double-blind, placebo-controlled trial. The Lancet Neurology. 2020;19(8):651-60.
31. Collaboration ET. Safety and efficacy of fluoxetine on functional recovery after acute stroke (EFFECTS): a randomised, double-blind, placebo-controlled trial. The Lancet Neurology. 2020;19(8):661-9.
